# Distinct dopaminergic spike-timing-dependent plasticity rules are suited to different functional roles

**DOI:** 10.21203/rs.3.rs-7456628/v1

**Published:** 2025-09-26

**Authors:** Baram Sosis, Jonathan E. Rubin

**Affiliations:** 1 Department of Mathematics, University of Pittsburgh, 301 Thackeray Hall, Pittsburgh, 15260, PA, USA.; 2 Present address: Berkeley, CA, USA.; 3 Center for the Neural Basis of Cognition, University of Pittsburgh, 4400 Fifth Ave, Pittsburgh, 15213, PA, USA.

**Keywords:** Dopamine, Synaptic plasticity, STDP, Basal ganglia, Reward prediction, Value estimation, Action selection

## Abstract

Various mathematical models have been formulated to describe the changes in synaptic strengths resulting from spike-timing-dependent plasticity (STDP). A subset of these models include a third factor, dopamine, which interacts with spike timing to contribute to plasticity at specific synapses, notably those from cortex to striatum at the input layer of the basal ganglia. Theoretical work to analyze these plasticity models has largely focused on abstract issues, such as the conditions under which they may promote synchronization and the weight distributions induced by inputs with simple correlation structures, rather than on scenarios associated with specific tasks, and has generally not considered dopamine-dependent forms of STDP. In this paper we introduce forms of dopamine-modulated STDP adapted from previously proposed plasticity rules. We then analyze, mathematically and with simulations, their performance in two biologically relevant scenarios. We test the ability of each of the three models to complete simple value estimation and action selection tasks, studying the learned weight distributions and corresponding task performance in each setting. Interestingly, we find that each plasticity rule is well suited to a subset of the scenarios studied but falls short in others. Different tasks may therefore require different forms of synaptic plasticity, yielding the prediction that the precise form of the STDP mechanism present may vary across regions of the striatum, and other brain areas impacted by dopamine, that are involved in distinct computational functions.

## 1 Introduction

Learning and memory are critical features of cognition and a number of neural learning mechanisms have been described. One important mechanism is spike-timing-dependent plasticity (STDP) [1, 2], a class of Hebbian plasticity rules in which the relative timing of pre- and postsynaptic spikes determines the changes in synaptic connection strength. Typically, a presynaptic spike before a postsynaptic spike – that is, a causal ordering of the spikes – leads to synaptic potentiation, whereas the reverse order leads to depression. In some cases, however, synaptic plasticity depends not just on the timing of pre- and postsynaptic spikes but also on some third factor, such as a neuromodulatory signal or other input [3, 4]. These additional factors may act as gating signals, and their strength and timing may impact both the magnitude and the direction of synaptic changes.

A prominent example of neuromodulatory impact on synaptic plasticity occurs at the cortical inputs to the basal ganglia. The neuromodulator dopamine is released by midbrain dopamine neurons when unexpected reward is received [5, 6] and plays a crucial role in modulating plasticity of corticostriatal synapses [7]. Experimental evidence and theoretical modeling suggest that dopamine serves as a reward prediction error signal [8, 9], enabling the brain to learn to favor behaviors that lead to reward and disfavoring behaviors that do not; such findings have been recently reviewed [10]. Theoretical analysis of action selection and modulation by cortico-basal ganglia-thalamic (CBGT) circuits posits a role for these dopaminergic reward prediction error signals both in updating value estimates associated with available choices and, through their impact on corticostriatal synaptic strengths, in altering the likelihood that a particular action will be selected in the future [11–14]. These distinct functions are likely performed by different neurons in different regions of the basal ganglia, however, which raises the possibility that distinct plasticity rules are involved. Unfortunately, despite some exciting experimental investigations of long-term plasticity properties in specific striatal regions and task settings [15–17], relatively little is known about the details of these plasticity mechanisms, especially in ventral striatal regions thought to encode value, which project to dopaminergic areas in the substantia nigra and hence are well positioned to contribute to calculation of reward prediction error [18, 19].

**Fig. 1:**
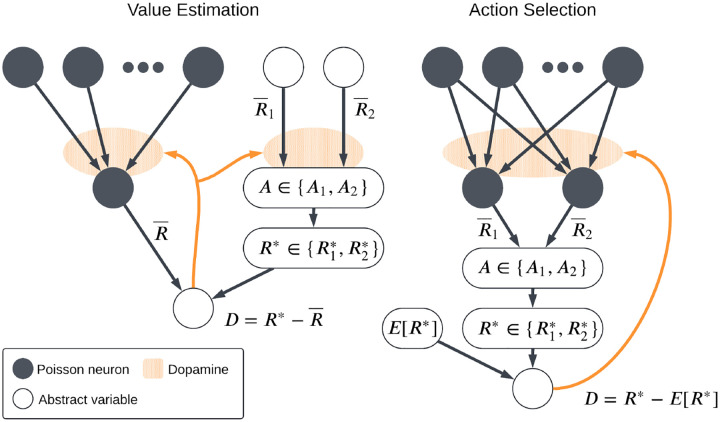
Schematic of the two main task settings. In the value estimation setting, the output firing rate $\overset{‾}{R}$ of the postsynaptic neuron is interpreted as a predicted reward and the dopamine signal D is the reward prediction error, while abstract variables $\left({\overset{‾}{R}}_{1},{\overset{‾}{R}}_{2}\right)$ are used to determine which action is selected and hence which reward $R^{*}$ is received. In the action selection setting, an action is chosen based on which of two competing channels has a higher output firing rate. A reward $R^{*}$ is then received based on the action taken and the dopamine D again represents reward prediction error, which depends on an abstract expected reward $E\left[R^{*}\right]$

Previous studies [14, 20, 21] have introduced computational models of reinforcement learning and action selection in the basal ganglia in which the strengths of the corticostriatal synapses determine the likelihood that each action will be selected. While these simulations employ biologically plausible neural models and synaptic plasticity rules, synaptic plasticity in these models is only used to learn action probabilities. Value estimates, in contrast, are modeled as abstract variables and modified using simple reinforcement learning rules. This raises the question of whether the same plasticity models used to learn action probabilities will also function well when used to learn action values, or whether different plasticity rules are needed for each task. To address this question, we consider two simple task settings, as illustrated in Figure 1. Both feature both action selection and value estimation, but we label them as the *action selection* and the *value estimation* settings, based on which part of the task is modeled using biologically plausible plasticity mechanisms rather than more abstract variables. Specifically, in the action selection setting, action probabilities are learned using explicit models of synaptic plasticity, while values are treated more abstractly; in the value estimation setting, action probabilities are treated abstractly while value estimates are learned using more explicit neural models. We then describe several models of learning based on STDP that incorporate dopaminergic modulation and evaluate their performance in both settings. We also test a variant of each setting in which the reward contingencies (i.e., which action leads to which reward) change periodically. We find that although each plasticity model does well in some cases, no model is able to succeed at both action probability learning and value learning. Thus, our results suggest that the brain, and in particular corticostriatal synapses in different regions of the striatum, may need to employ distinct, specialized plasticity mechanisms to learn different tasks.

## 2 Models

### 2.1 Plasticity Models

#### 2.1.1 General Modeling Framework

Here we introduce several models of dopaminergic spike-timing-dependent plasticity. The *additive model* is based on incorporating dopamine into existing models of STDP that had been previously considered without dopamine [22, 23]. Our description and analysis of this model mainly follows the presentation in [24]. A plasticity rule known as the *multiplicative model* [25–27] was proposed to correct some deficiencies in the additive model; for reasons described below, we do not use this model, instead introducing an updated version of this plasticity rule that we call the *symmetric model*, which is better suited to modeling dopaminergic plasticity. We also study a third model, what we call the *corticostriatal model*, based on a computational model of plastic corticostriatal synaptic connections, specifically connections onto striatal spiny projection neurons that express the D1 dopamine receptor, sometimes referred to as direct pathway SPNs, as described in [21]. This model incorporates recent experimental findings about synaptic plasticity and eligibility traces in these neurons [28–34] and builds on other recent modeling studies [11–14].

We consider linear Poisson neurons: presynaptic spike trains are modeled as Poisson processes $\rho_{i}^{\text{pre}}(t)$ with constant rate $r_{i}={\left\langle \rho_{i}^{\text{pre}}(t)\right\rangle }_{t}$ (where i=1,2,…,N), and likewise the spike train of each postsynaptic neuron in each model is a Poisson process $\rho^{\text{post}}(t)$ with instantaneous firing rate function R(t) given by a linear combination of the presynaptic spike trains

(1) $$R(t)=\frac{1}{N}\sum_{i=1}^{N}w_{i}(t)\rho_{i}^{\text{pre}}(t-\epsilon )$$

where ϵ>0 is a small synaptic delay and $w_{i}$ are the synaptic efficacies, which we will also call weights, normalized to lie in [0, 1]. We will write the vectors of input firing rates and weights as $r,w\in R^{N}$. This set-up can be implemented in simulations by first generating input spike trains $\rho_{i}^{\text{pre}}(t)$, and then, whenever a presynaptic spike from input unit i occurs, say at time $t_{\text{pre}}$, adding a postsynaptic spike at time $t_{\text{post}}=t_{\text{pre}}+\epsilon$ to the postsynaptic spike train $\rho^{\text{post}}$ with probability $w_{i}\left(t_{\text{pre}}\right)/N$. We assume that the input spike trains are uncorrelated. Note that in some cases we will have two postsynaptic neurons; then, each will have its own spike train, firing rate function, and so on, but since the two do not interact and hence can be treated independently, we simplify the notation by omitting subscripts for postsynaptic quantities.

In general terms, to implement synaptic plasticity, or changes in the weights, that depends on dopamine, rather than modifying each synapse immediately with the occurrence of every spike pair, as in a classical two-factor STDP rule, we instead define eligibility traces [35, 36] to track spike pairs. The eligibility traces for each synapse decay exponentially between spike pairs and are incremented when the pre- and post-synaptic neurons associated with a synapse both spike in a certain order. Weight changes are proportional to both the current values of the eligibility traces and the value of a dopamine signal, which we also model.

At a more detailed level, we base our implementation of this model on the procedure described in [14], which uses a set of trace variables to track the influences of pre- and postsynaptic spikes and spike pairs. Specifically, we define $A_{i}^{\text{pre}}(t)$ and $A^{\text{post}}(t)$ to track the pre- and postsynaptic spiking,

(2) $$\frac{dA_{i}^{\text{pre}}}{dt}=\rho_{i}^{\text{pre}}\left(t\right)-\frac{1}{\tau }A_{i}^{\text{pre}}\left(t\right),\;\frac{dA^{\text{post}}}{dt}=\rho^{\text{post}}\left(t\right)-\frac{1}{\tau }A^{\text{post}}\left(t\right),$$

with decay constant τ>0. An important assumption in our analysis, made by [24, 26] and others, is that changes in weight from individual spike pairs can be summed independently. To realize these changes, we define two eligibility traces, $E_{i}^{+}(t)$ and $E_{i}^{-}(t)$, to track pre-before-post and post-before-pre spike pairs, respectively,

(3) $$\frac{dE_{i}^{+}}{dt}=\rho^{\text{post}}(t)A_{i}^{\text{pre}}(t)-\frac{1}{\tau_{\text{eli}}}E_{i}^{+}(t)\;\frac{dE_{i}^{-}}{dt}=\rho_{i}^{\text{pre}}(t)A^{\text{post}}(t)-\frac{1}{\tau_{\text{eli}}}E_{i}^{-}(t),$$

with decay constant $\tau_{eli}>0$. We use two independent traces in part because experimental results have suggested that this independence is present in cortical synapses [37]. Moreover, using a single trace, as done previously [14], allows spike pairs to interact nonlinearly and partially cancel each other out, while using two traces ensures that different spike pairs do not interact, which is convenient for analysis. In Appendix D we show that using a modified plasticity model with a single eligibility trace gives qualitatively similar results in most cases, and does not meaningfully improve performance on the tasks we study here.

We assume for simplicity that dopamine is released periodically at fixed intervals of length $1/r^{\text{dop}}$ for constant $r^{\text{dop}}>0$; otherwise, it decays exponentially:

$$\frac{dD}{dt}=\sum_{k}D_{k}\delta \left(t-k/r^{\text{dop}}\right)-\frac{1}{\tau_{\text{dop}}}D.$$

The value of the dopamine increment $D_{k}$ depends on the task setting; see Section 2.2. We will treat this signal as the dopamine level *relative to some baseline*, rather than the raw dopamine concentration itself; so, in the absence of any signal, D equals zero, and we allow $D_{k}<0$. Note that while the dopamine *concentration* may depend on the postsynaptic spike train, we assume for analytical convenience that the *timing* of dopamine delivery is independent of the spiking activity. The precise form of the dopamine process is irrelevant as long as it has mean rate $r^{\text{dop}}$, is independent of the spike trains, and yields dopamine signals that are far enough apart that their interactions can be neglected.

Finally, the weights in equation (1) are updated using the values of the dopamine signal and the eligibility traces in a way that depends on the choice of plasticity model. The additive, multiplicative, and symmetric models use the following rule:

(4) $$\frac{dw_{i}}{dt}=\lambda D\left(t\right)\left(f_{+}\left(w_{i}\left(t\right)\right)E_{i}^{+}\left(t\right)-f_{-}\left(w_{i}\left(t\right)\right)E_{i}^{-}\left(t\right)\right),$$

where $f_{+}$ and $f_{i}$ depend on the plasticity model used and λ>0 is the learning rate. The corticostriatal model is formulated somewhat differently, as described in Section 2.1.3.

#### 2.1.2 Additive and Multiplicative Models

For the additive model, $f_{+}(w)=1$ and $f_{-}(w)=\alpha$, where α tunes how strongly negative eligibility is weighted relative to positive eligibility (typically α≥1). This model has the advantage of simplicity; however, because $f_{\pm }$ are independent of w, it allows the weights to change without bound. In past implementations of this model, the weights were artificially constrained between 0 and 1 to avoid this issue, but a model that keeps the weights bounded naturally due to its inherent dynamics would likely be a more realistic model of synaptic plasticity. This led to the development of the multiplicative model [25–27], in which $f_{+}(w)=1-w$ and $f_{-}(w)=\alpha w$. (See [24] for an exploration of models of the form $f_{+}(w)=(1-w{)}^{\mu },f_{-}(w)=\alpha w^{\mu }$ where μ∈[0,1] interpolates between the additive and multiplicative models.) In previous studies, which did not include the dopamine term D(t) in equation (4), the multiplicative model kept the weights bounded between 0 and 1: the 1-w term shrinks positive updates from $E^{+}$ near the upper boundary, while the αw term shrinks negative updates from $E^{-}$ near the lower boundary. However, this bounding breaks down with the introduction of dopamine. If D(t) is negative, then the $-D(t)E_{i}^{-}(t)$ term will be positive, so the αw scaling factor will not prevent the weights from going above 1; similarly, the $D(t)E_{i}^{+}(t)$ term will be negative, for which a 1-w scaling factor is inappropriate for maintaining positivity of weights. The multiplicative model is therefore not effective at constraining the weights in our setting, and hence we do not consider it in our analysis.

#### 2.1.3 Alternative Models: Symmetric and Corticostriatal

One solution to this weight bounding problem is given by what we term the symmetric model, which uses $f_{+}(w)=w(1-w)$ and $f_{-}(w)=\alpha w(1-w)$. (This adjustment is identical to multiplying the dynamical equations for the additive model by w(1-w).) This choice ensures that no matter the sign of the dopamine signal or the eligibility, the weight updates will be scaled to prevent them from going past 0 or 1, as both positive and negative updates are multiplied by the same term, w(1-w). However, whether the scaling factor α applies to potentiation or depression in this formulation still depends on the sign of the eligibility, and not on the dopamine.

An alternative solution, which we refer to as the corticostriatal model, is to tie the functional form of the weight update to the sign of the overall weight change, rather than just to the sign of the eligibility. In other words, we use a scaling factor of 1-w when the sign of the weight change (taking into account the sign of the dopamine signal and the sign of the eligibility) is positive, and αw when it is negative. This convention is described by the formula

(5) $$\frac{dw_{i}}{dt}=\left\{\begin{array}{ll} \lambda D(t)\left(\left(1-w_{i}(t)\right)E_{i}^{+}(t)-\alpha w_{i}(t)E_{i}^{-}(t)\right) & \text{if}\;D(t)\geq 0,\\ \lambda D(t)\left(\alpha w_{i}(t)E_{i}^{+}(t)-\left(1-w_{i}(t)\right)E_{i}^{-}(t)\right) & \text{if}\;D(t)<0. \end{array}\right.$$

Table 1 shows how the scaling factors used by each of the four models depend on the signs of the dopamine and eligibility. The additive, multiplicative, and symmetric models only depend on the sign of the eligibility, with one factor used when $E_{i}<0$ and another when $E_{i}>0$, while the corticostriatal model uses the sign of the product of dopamine and eligibility to determine which scaling factor to use. Thus, in the corticostriatal model, the scaling factor corresponds to the direction in which the weights will change: 1-w for increasing weights and αw for decreasing weights. In contrast, the scaling factors used by the other models do not correspond to the direction of weight change.

To summarize, the additive model allows the weights to grow without bound (unless they are artificially constrained); the multiplicative model does not effectively solve this problem when dopamine is incorporated into the model, while the symmetric and corticostriatal models offer two different ways of effectively constraining the weights. For the remainder of this paper we focus on the additive, symmetric, and corticostriatal models.^1^ The corticostriatal model appears to be the most natural one to use in modeling corticostriatal synaptic plasticity, and it has been employed in recent modeling work [21]. As we show below, in a setting where the synaptic weights govern the likelihood that particular actions will be selected, the corticostriatal model performs well. However, it does not perform well when the weights govern value estimation. In such settings a different plasticity rule may yield an improved outcome.

Importantly, in all models, synapses become stronger with above-baseline dopamine signals (and weaker with below-baseline dopamine signals) when the post-synaptic neuron has recently participated in a pre-before-post spike pairing, and weights change in the opposite direction following post-before-pre spike pairs. These properties are implemented to match the observed behaviors of cortical synapses onto striatal spiny projection neurons expressing specifically D1 dopamine receptors [11, 13, 14, 21, 29, 32]. Neurons expressing D2 receptors show the opposite behavior, but we do not consider those here.

### 2.2 Task Settings

We consider two main task settings, corresponding to two different functional roles that striatal neurons may play: *action selection* and *value estimation*. In both the action selection and value estimation settings, the dynamics of eligibility and synaptic plasticity are as described in Section 2.1, with the dopamine signals given by equations (8) and (10), respectively.

#### 2.2.1 Action Selection Setting

In the action selection setting, we explicitly model the plasticity mechanisms that govern action selection. We implement this as a competition between two action channels [20, 38–40]. Two neurons with weight vectors $w^{1}$ and $w^{2}$ (with entries $w_{i}^{j}$ for i∈{1,2,…,N} and j∈{1,2}) receive individual, statistically independent input spike trains, with firing rates given by the same firing rate vector r for both $w^{1}$ and $w^{2}$, modeling a situation in which the two channels receive the same information from different upstream sources. A natural extension of this setting would be to employ correlated inputs, corresponding to shared presynaptic input sources, but for simplicity we do not consider this here. We compute estimates of their current firing rates ${\overset{‾}{R}}_{1},{\overset{‾}{R}}_{2}$ as follows:

(6) $${\overset{‾}{R}}_{j}=\frac{1}{T_{\text{win}}}\int_{t_{\text{dop}}-T_{\text{win}}-T_{\text{del}}}^{t_{\text{dop}}-T_{\text{del}}}\rho_{j}^{\text{post}}\left(t\right)dt.$$

**Fig. 2:**
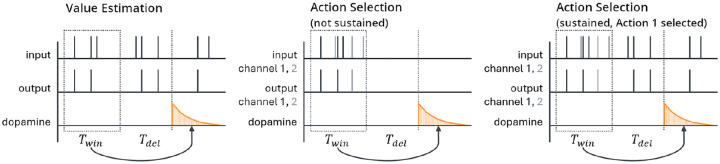
Schematic of the sequence of events in the value estimation (left) and action selection (center, right) settings. Output spikes are counted in a window of length $T_{\text{win}}$ to estimate the average output firing rate; then, after a delay of length $T_{\text{del}}$, dopamine is released. For the action selection setting there are two channels, colored black and gray, corresponding to the two actions being considered. In the action selection setting, we generally sustain activity in the selected channel while suppressing activity in the other channel (right), but we also test a version where activity is suppressed in both channels (center) while varying $T_{\text{del}}$

Here $T_{\text{win}}$ is the length of the time window over which the spike train is averaged and $T_{\text{del}}$ is a delay term between when the output firing rate is measured and the time $t_{\text{dop}}$ when the dopamine is actually released (see Figure 2). This delay could be due to biological constraints, such as the speed of neural signal propagation or motor response, or to experimentally imposed delays.

The agent in this model probabilistically chooses one of two actions, $A_{1}$ or $A_{2}$, with selection probabilities based on the output firing rates:

(7) $$p=P\left(A=A_{1}\right)=\frac{e^{\beta {\overset{‾}{R}}_{1}}}{e^{\beta {\overset{‾}{R}}_{1}}+e^{\beta {\overset{‾}{R}}_{2}}}$$

where β is an inverse temperature parameter and $P\left(A=A_{2}\right)=1-p$. (For simplicity, in simulations in this setting we take β to be an arbitrary large number, so that actions are chosen deterministically based on which channel has more spikes, with ties broken randomly.) The agent receives a reward $R^{*}$ depending on which action is taken: $R^{*}=R_{1}^{*}$ if $A_{1}$ is chosen and $R^{*}=R_{2}^{*}$ if $A_{2}$ is chosen. Finally, the dopamine signal is computed as the reward prediction error,

(8) $$D=R^{*}-E\left[R^{*}\right]=R^{*}-\left(R_{1}^{*}E[p]+R_{2}^{*}(1-E[p])\right),$$

which is used to update the synaptic weights and hence $\rho_{j}^{\text{post}}(t)$ and ${\overset{‾}{R}}_{j}$, thus impacting future action selection. Note that in this setting we do not model the neural mechanisms that implement value estimation, instead simply computing p and D explicitly. A successful plasticity model in this task setting is one that can learn to more frequently take the action that gives the higher reward.

In equation (8), p is treated as a random variable; we take the average value of p over instantiations of the spike trains with the given rates. That is, to compute E[p], we sum over the possible postsynaptic spike counts in each channel,

(9) $$E[p]=\sum_{i=0}^{\infty }\sum_{j=0}^{\infty }\frac{e^{i\beta /T_{\text{win}}}}{e^{i\beta /T_{\text{win}}}+e^{j\beta /T_{\text{win}}}}\frac{n_{1}^{i}e^{-n_{1}}}{i!}\frac{n_{2}^{j}e^{-n_{2}}}{j!},$$

where

$$n_{k}=T_{\text{win}}\frac{\left\langle w^{k},r\right\rangle }{N},\;k\in \{1,2\}$$

is the expected number of postsynaptic spikes in a window of length $T_{\text{win}}$.

This definition assumes that the agent’s state-value function [36] is accurate. In other words, the agent has learned the reward it receives on average when performing this task with its current policy (as defined by the weights $w^{1}$ and $w^{2}$). The idea that value estimates are available to neurons that drive action selection is commonly used in models and has ample experimental support (e.g., [41, 42]). In practice these value estimates have to be learned, and as the animal’s policy changes, the value estimates will have to evolve along with it. In the action selection setting, we assume that the value estimates remain accurate (i.e., are learned instantaneously relative to the timescale of decision policy changes) as a simplification to allow us to focus on the action selection task without the added complication of a separate value learning circuit.

We silence all input to the striatal neuron between the end of one spike count window (used in equation (6)) and the beginning of the next (i.e., for the duration of the delay if $T_{\text{del}}\neq 0$ as well as the period after dopamine is released before the next spike count window starts, which depends on $r^{\text{dop}}$, the rate at which dopamine is released). This step is designed to represent typical experimental settings in which the input stimulus does not persist after an action is taken in response to the stimulus. For instance, in a task in which a rodent must choose which branch of a maze to follow to receive a reward, the stimulus – the sight of the junction – necessarily cannot persist after the animal has made a choice and gone down one of the branches. However, if $T_{\text{del}}$ is large compared to $\tau_{\text{eli}}$ (equation (3)), then the eligibility will be approximately zero by the time dopamine is released, leading to minimal weight changes. We therefore generally suppose that the cortex maintains some level of activity in the channel corresponding to the selected action [43, 44] to help correctly assign credit for rewards to actions when they are separated by significant delays. Specifically, we suppose that during the interval between spike count windows, the firing rates in the selected channel equal $a_{\text{sel}}r_{i}$ for some $a_{\text{sel}}\in (0,1]$, and are zero in the channel that was not selected. We also briefly test a version of the model without this sustained activity. Figure 2 shows an illustration of the two versions of the action selection setting as well as a comparison to the value estimation setting (described below).

#### 2.2.2 Value Estimation Setting

The second task setting that we consider is what we will call the *value estimation setting*. Whereas in the action selection setting we explicitly link action selection to a comparison of postsynaptic (i.e., striatal) firing rates and leave the value estimation process fairly abstract, here we do the reverse, modeling the neural implementation of value estimation while leaving action selection abstract. We use a single postsynaptic neuron with weight vector w; as before, it receives independent input spike trains from a collection of presynaptic neurons with firing rates specified by entries in the vector r. We estimate the postsynaptic firing rate $\overset{‾}{R}$ as in equation (6); in this setting, this rate represents the estimated value. To select actions, we track two variables, ${\overset{‾}{R}}_{1}$ and ${\overset{‾}{R}}_{2}$, representing how much each action is favored. These are not computed by estimating the firing rate of some neurons as in the action selection setting. They may be taken to represent the activity level of some neural population, but in the model they are simply treated as abstract variables. (Their dynamics will be described below.) We compute the probability of taking each action using equation (7) as before, and the agent receives a reward $R^{*}$ with value $R_{1}^{*}$ or $R_{2}^{*}$ depending on whether action $A_{1}$ or $A_{2}$ is selected. The dopamine signal is then given by

(10) $$D=R^{*}-\overset{‾}{R},$$

which is used to update the synaptic weights w using one of the plasticity rules described previously.

The dopamine signal is also used to update the values of ${\overset{‾}{R}}_{1}$ and ${\overset{‾}{R}}_{2}$. We assume that changes in these variables are proportional to the magnitude of the dopamine signal. We also assume that if action $A_{1}$ is selected and the dopamine signal is positive, then ${\overset{‾}{R}}_{1}$ will increase and ${\overset{‾}{R}}_{2}$ will decrease, and vice versa if dopamine is negative or if $A_{2}$ is selected. To model this, we define the variable $\overset{‾}{A}(t)$ as 1 if the previous action was $A_{1}$ and −1 if the previous action was $A_{2}$. Then ${\overset{‾}{R}}_{1}$ and ${\overset{‾}{R}}_{2}$ obey the following dynamical equation:

(11) $$\frac{d{\overset{‾}{R}}_{j}}{dt}=(-1{)}^{j-1}\frac{\overset{‾}{\lambda }}{2}D(t)\overset{‾}{A}(t)$$

where $\overset{‾}{\lambda }$ is a learning rate. (We include a factor of 1/2 here to eliminate a factor of 2 that arises later in our analysis.) Note that tracking both ${\overset{‾}{R}}_{1}$ and ${\overset{‾}{R}}_{2}$ is redundant, as it can be shown from equation (7) that the probability of picking each action only depends on their difference. Our simulations use a single variable, ${\overset{‾}{R}}_{\text{diff}}={\overset{‾}{R}}_{1}-{\overset{‾}{R}}_{2}$, but for our mathematical analysis we find it convenient to express the system in terms of p, the probability of picking action $A_{1}$ (see Appendix A).

Note that as there is only one channel in the value estimation setting, we do not reduce its activity level outside the spike count window using $a_{\text{sel}}$ like we do in the action selection setting and instead simply keep the input firing rate constant.

### 2.3 Simulations

We use the parameter values listed in Table 2 as the defaults in our simulations for each of the two main task settings; any other parameters or changes to the defaults are listed in figure captions. “Steps” refers to the number of cycles of dopamine release in each simulation; this number as well as the learning rates λ and $\overset{‾}{\lambda }$ were chosen to balance noise level with computation time and to illustrate phenomena of interest. $w_{\text{init}}=0.5$ and $p_{\text{init}}=0.5$ were chosen because they lie at the midpoint of [0, 1] and hence represent unbiased starting values. We chose input firing rates r to roughly match the frequency of cortical input to the striatum. $R_{1}^{*}$ and $R_{2}^{*}$ are arbitrary and were chosen for illustrative purposes. We use α=1 as the default scaling parameter in our weight update equations (equations (4) and (5)) for simplicity. For the choice of τ=0.02s in equation (2), see [2] and [24]; note that [45] give values of τ=0.0168s for the decay constant of long-term potentiation (LTP) as a function of pre-before-post spike separation and τ=0.0337s as the analogous, post-before-pre quantity for long-term depression (LTD), but as we use τ to track the window of influence for both pre- and post-synaptic spikes, we use the intermediate value of τ=0.02s used in other sources. The half-life of dopamine has been estimated as 0.72 s in the dorsolateral striatum [46]; translating the half-life into an exponential time constant we get $\tau_{\text{dop}}\approx 1\text{s}$. The choice of eligibility time constant $\tau_{\text{eli}}=1\;\text{s}$ reflects experimentally derived estimates ([33, 47]; but see [34], which finds a somewhat larger value). The delay time for dopamine release, $T_{\text{del}}$, was chosen to be long enough that any spikes occurring before the delay have minimal impact on the weight changes. $r^{\text{dop}}$ was likewise chosen to be small enough that the effects of any interactions between adjoining dopamine signals would be negligible. The models appear more sensitive to these parameters in the action selection setting, necessitating longer delays. The constant β was selected arbitrarily. In the value estimation setting we use β=1; in the action selection setting we use $\beta =10^{6}$ to make the choice essentially deterministic for a given set of spike counts, for simplicity. The parameter $a_{\text{sel}}$ was set to 0.7 to match the corresponding parameter “sustainedfraction” in [21].

All figures use a sample size of 100 trials for numerical results besides Figure 10 and Figure 11, which use 10000; error bars and bands show standard deviations. In some phase portraits we include critical points; these were found analytically when possible, otherwise they were computed using the scipy.optimize library [48]. Note that some of the critical points found on the boundaries are not “true” critical points in the sense that they are not zeros of the dynamical equations. Rather, they are the result of the weights being clipped at 0 and 1. All code needed to run the simulations and reproduce the figures in the paper is available on GitHub at https://github.com/bsosis/DA-STDP.

## 3 Results

### 3.1 Action Selection Setting

#### 3.1.1 Analysis of Dynamics

We first consider a task of selecting between two actions in which the probability of picking each action is governed by one of the three plasticity rules, as described in Section 2.2. In Appendix C.1 we show that under suitable assumptions it is possible to derive a formula for the average drift of the weights over time for the additive and symmetric models in this setting:

(12) $${\dot{w}}_{i}^{j}=(-1{)}^{j-1}\left(R_{1}^{*}-R_{2}^{*}\right)E[p](1-E[p])r^{\text{dop}}\tau_{\text{dop}}\tau_{\text{eli}}\frac{\lambda }{N}\times \left(a_{\text{sel}}^{2}\tau \left\langle w^{j},r\right\rangle \Delta f\left(w_{i}^{j}\right)r_{i}+a_{\text{sel}}f_{+}\left(w_{i}^{j}\right)w_{i}^{j}r_{i}\right)\;$$

where $\Delta f=f_{+}-f_{-}$ and E[p] is given by equation (9). An important assumption made in this derivation is that the delay $T_{\text{del}}$ is large relative to $\tau_{\text{eli}}$; this assumption will be discussed below. We can interpret the terms in equation (12) as follows. The $\tau \left\langle w^{j},r\right\rangle \Delta f\left(w_{i}^{j}\right)r_{i}$ term corresponds to independent pairs of pre- and postsynaptic spikes (both pre-before-post and post-before-pre), while the $f_{+}\left(w_{i}^{j}\right)w_{i}^{j}r_{i}$ term corresponds to a pre-post spike pair in which the presynaptic spike directly causes the postsynaptic neuron to fire. Recall that when action $A_{k}$ is selected, activity in channel k is reduced by a factor of $a_{\text{sel}}$, while activity in the other channel is suppressed entirely. To compute ${\dot{w}}_{i}^{j}$ we therefore condition on action $A_{j}$ being selected, multiplying the expression by either E[p] or 1-E[p] (for actions $A_{1}$ or $A_{2}$, respectively), and we multiply the firing rates $r_{i}$ in the above expression by $a_{\text{sel}}$. Finally, it can be shown that the average dopamine signal, conditioned on either action $A_{1}$ or $A_{2}$ being selected, is $\left(R_{1}^{*}-R_{2}^{*}\right)(1-E[p])$ or $\left(R_{2}^{*}-R_{1}^{*}\right)E[p]$, respectively. This gives the above expression.

In general, if $R_{1}^{*}>R_{2}^{*}$ as we typically assume, then we would like the difference between the weights, $w^{1}-w^{2}$ (or $\left\langle w^{1},r\right\rangle -\left\langle w^{2},r\right\rangle$ if N>1), to be positive and as large as possible, so that the probability of picking action $A_{1}$ is maximized. Since the weights are bounded by 0 and 1, this corresponds to the point $w_{i}^{1}=1,w_{i}^{2}=0$ for all i. If $R_{1}^{*}<R_{2}^{*}$ then the optimal set of weights is at $w_{i}^{1}=0,w_{i}^{2}=1$. Equation (12) always has w=0, where $0=(0,\ldots ,0)\in R^{N}$, as an equilibrium. For the symmetric model, it additionally has an equilibrium at w=1, where $1=(1,\ldots ,1)\in R^{N}$. (When N>1 the symmetric model also has a number of other equilibria, described in Appendix C.2, which we will not analyze further.) In Appendix C.2 we prove the following result about the dynamics of these models:

**Theorem 1** *Suppose that for the additive or symmetric models in the action selection setting, the following condition is satisfied:*

(13) $$a_{sel}\tau \left(\alpha -1\right)<\frac{1}{\|r{\|}_{1}},$$

*and suppose* $R_{1}^{*}>R_{2}^{*}$. *Then the* $w^{1}=w^{2}=0$ *equilibrium is a saddle that is repelling along the* $w^{1}$ *coordinates and attracting along the* $w^{2}$ *coordinates. For the symmetric model*, $w^{1}=1,w^{2}=0$ *is an attractor*, $w^{1}=0,w^{2}=1$ *is a repelling equilibrium, and* $w^{1}=w^{2}=1$ *is a saddle that is attracting along the* $w^{1}$ *coordinates and repelling along the* $w^{2}$ *coordinates*.

These relationships are reversed if $R_{1}^{*}<R_{2}^{*}$. In both cases, if equation (13) holds, the flow is in the direction of the optimal set of weights for the task: $w_{i}^{1}=1,w_{i}^{2}=0$ for all i if $R_{1}^{*}>R_{2}^{*}$ and $w_{i}^{1}=0,w_{i}^{2}=\text{for all}i$ if $R_{1}^{*}<R_{2}^{*}$.

The corticostriatal model is somewhat more complex to analyze. In Appendix C.1 we derive the following system of equations describing its average weight drift, which holds when $R_{1}^{*}>R_{2}^{*}$:

(14) $${\dot{w}}_{i}^{1}=|R_{1}^{*}-R_{2}^{*}|E\left[p\right]\left(1-E\left[p\right]\right)r^{\text{dop}}\tau_{\text{dop}}\tau_{\text{eli}}\frac{\lambda }{N}\times \left(a_{\text{sel}}^{2}\tau \left\langle w^{1},r\right\rangle \left(1-\left(1+\alpha \right)w_{i}^{1}\right)r_{i}+a_{\text{sel}}\left(1-w_{i}^{1}\right)w_{i}^{1}r_{i}\right),\;{\dot{w}}_{i}^{2}=|R_{1}^{*}-R_{2}^{*}|E\left[p\right]\left(1-E\left[p\right]\right)r^{\text{dop}}\tau_{\text{dop}}\tau_{\text{eli}}\frac{\lambda }{N}\times \left(a_{\text{sel}}^{2}\tau \left\langle w^{2},r\right\rangle \left(1-\left(1+\alpha \right)w_{i}^{2}\right)r_{i}-a_{\text{sel}}\alpha {\left(w_{i}^{2}\right)}^{2}r_{i}\right).$$

If $R_{1}^{*}<R_{2}^{*}$, then we obtain an analogous system with the forms of the right hand sides swapped. In addition to the equilibrium point with all w components equal to zero, these equations have an extra equilibrium at the point $\left(w^{1*},w^{2*}\right)$ defined by

$$w_{i}^{1*}=\frac{a_{\text{sel}}\tau \|r{\|}_{1}+1}{a_{\text{sel}}\tau \|r{\|}_{1}(1+\alpha )+1}\;\forall i,$$

$$w_{i}^{2*}=\frac{a_{\text{sel}}\tau \|r{\|}_{1}}{a_{\text{sel}}\tau \|r{\|}_{1}(1+\alpha )+\alpha }\;\forall i.$$

There are also equilibria at the points $\left(0,w^{2*}\right)$ and $\left(w^{1*},0\right)$.

We show in Appendix C.2 that both $w^{1*}$ and $w^{2*}$ are attractors for their respective equations, so that $w^{1}$ converges to $w^{1*}$ and $w^{2}$ converges to $w^{2*}$. Since $w^{1*}>w^{2*}$, the more rewarding action, $A_{1}$, will be favored over $A_{2}$, although the difference in weights after convergence may not be as great as that achievable under the additive or symmetric models.

#### 3.1.2 Simulation Results

**Fig. 3:**
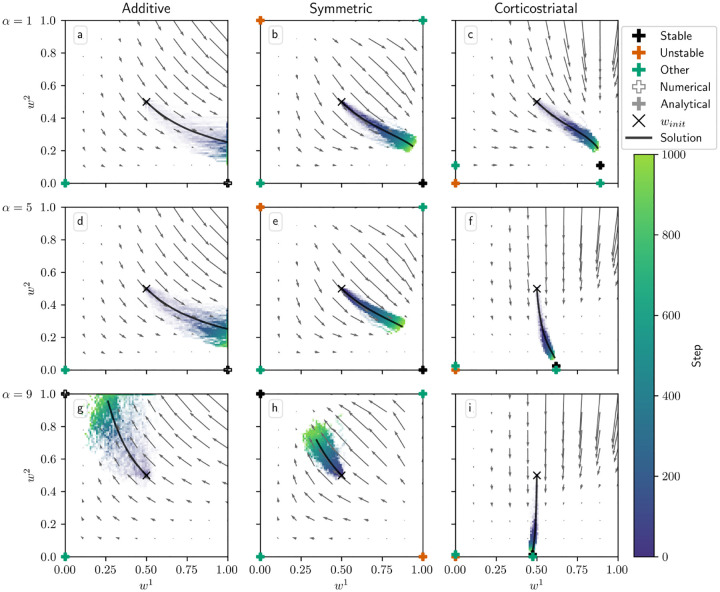
Distribution of $w^{1}$ and $w^{2}$ over time in the action selection setting as α is varied. The color code indicated in the color bar indicates the simulation step. Columns show the additive (a, d, g), symmetric (b, e, h), and corticostriatal (c, f, i) models. α is varied across rows: (a-c) α=1; (d-f) α=5; (g-i) α=9. Each panel includes the model equilibria as well as arrows showing the vector field of the averaged model. Note that the vector field arrows are scaled to highlight relative flow intensities within individual plots; this scaling varies across plots. Orange coloring of the equilibria indicates unstable critical points; black indicates stable; green indicates any other equilibria. Filled markers indicate that the critical point was found analytically; empty markers indicate those found numerically. We use $w_{\text{init}}=0.5$ here, marked by the “×“ in each plot. The numerical solution to the averaged system starting at $w_{\text{init}}=0.5$ is plotted in gray

Figure 3 shows phase portraits for the models for three different values of α for N=1, i.e. a single weight per channel. To generate each plot, we ran a set of simulations of the fully stochastic (i.e., non-averaged) implementation (see Section 2.3) of the appropriate model with initial condition $w_{\text{init}}=(0.5,0.5)$ as marked by the × symbol. In each simulation, from this starting point, $w^{1}$ and $w^{2}$ evolved over 1000 time steps, and the positions of $\left(w^{1},w^{2}\right)$ at certain time steps were plotted as points with time-dependent coloring as indicated by the color bar; this process resulted in a cloud of points over many simulations, representing the distribution of weights over time. Each plot also includes all critical points, the actual trajectory from $w_{\text{init}}$, and the vector field arrows for the averaged model. The orientations of these arrows indicate the directions that trajectories would move over time, from various starting positions, under the flow of the averaged model, while their lengths represent the magnitudes of the weights’ rates of change.

We can see that the behavior of the system matches what we described above. Since $R_{1}^{*}>R_{2}^{*}$, when α is not too large, under the additive and symmetric models, $w^{1}$ increases while $w^{2}$ decreases, leading to a high probability of selecting the more rewarding action, $A_{1}$. However, when α crosses the threshold defined by equation (13), in this case α≈8.14, the signs of the dynamical equations switch, and the system no longer selects the more-rewarded action (Figure 3g–h). Meanwhile, the weights under the corticostriatal model converge to the $w^{1*}$ and $w^{2*}$ equilibria defined above. Since $w^{1*}>w^{2*}$, this leads to selection of action $A_{1}$ more often than action $A_{2}$ regardless of α (although the precise probability of selecting each action converges to a value that does depend on α).

Note that in the plots based on the additive model there are extra critical points at $w^{1}=1,w^{2}=0$ (for α=1,5) and $w^{1}=0,w^{2}=1$ (for α=9). These are not “true” critical points in the sense that they are not zeros of the dynamical equations. Rather, they are accumulation points resulting from the clipping of the weights at 0 and 1. Such points were found numerically, and are indicated with unfilled markers (see Figure 3 legend).

**Fig. 4:**
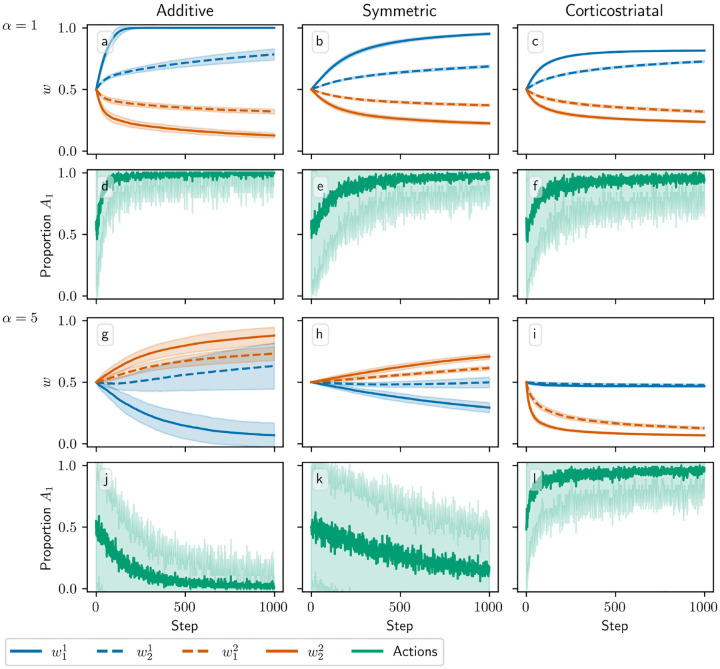
Model performance in the action selection setting as α is varied for N=2. Plots show weights (a-c,g-i) and proportion of trials in which action $A_{1}$ is selected (d-f,j-l) versus time for the additive (a, d, g, j), symmetric (b, e, h, k), and corticostriatal (c, f, i, l) models. α is varied across rows: (a-f) α=1; (g-l) α=5. Here $r=(15,5)\;{\text{s}}^{-1}$

In Figure 4 we plot the behavior of the system when N=2. In this case there are four weights to consider: $w^{1}=\left(w_{1}^{1},w_{2}^{1}\right)$ and $w^{2}=\left(w_{1}^{2},w_{2}^{2}\right)$. We use $r=(15,5)\;{\text{s}}^{-1}$. The optimal weight values are still at $w^{1}=(1,1)$ and $w^{2}=(0,0)$, but the asymmetry between $r_{1}$ and $r_{2}$ leads to different rates of convergence. We can see in Figure 4 that when α=1, the behavior of the three models is qualitatively similar to that in Figure 3. When α=5, though, the additive and symmetric models fail to converge to the appropriate equilibrium, because condition (13) is no longer satisfied (the condition fails at α≈4.57 for these parameter values). Meanwhile, the corticostriatal model continues converging to $w^{1*}$ and $w^{2*}$, and since $w^{1*}>w^{2*}$, this leads to selecting the correct action.

**Fig. 5:**
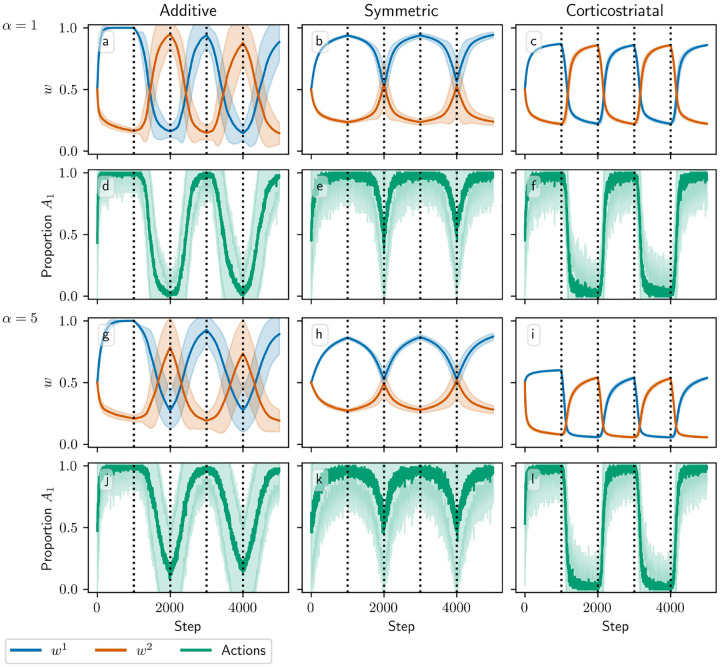
Model performance in the action selection setting with contingency switching as α is varied. Plots show weights (a-c, g-i) and proportion of trials in which action $A_{1}$ is selected (d-f, j-l) versus time for the additive (a, d, g, j), symmetric (b, e, h, k), and corticostriatal (c, f, i, l) models. α is varied across rows: (a-f) α=1; (g-l) α=5. Here $r=10{\;\text{s}}^{-1}$ and the reward contingencies switch between $R_{1}^{*}=2,R_{2}^{*}=1$ and $R_{1}^{*}=1,R_{2}^{*}=2$ every 1000 steps

Figures 3 and 4 show results for learning of a single relation between action and reward. In some situations, both in experiments and in natural settings, relations between actions and subsequent rewards can change over time, an effect that we refer to as contingency switching. To simulate this scenario, we swap which action is mapped to the higher reward value every 1000 steps. With this adjustment, we find that substantial differences arise in performance among the three models, as shown in Figure 5. For α=1, the corticostriatal model is able to quickly react to the contingency switches and swap which action it takes, resulting in only brief drops in accuracy when switches occur. The additive model is also able to swap which action it takes, although it is significantly slower to do this and less consistent than the corticostriatal model. The symmetric model, however, is unable to perform this task well, because the weights become stuck near the widely spread values that they attain for the first contingency scenario. Increasing α alters the behavior of the models somewhat, but for these parameter values the corticostriatal model is able to maintain good performance.

We can understand these differences in performance by referring to equations (12) and (14) and Figure 3. Swapping $R_{1}^{*}$ and $R_{2}^{*}$ has the effect of mirroring the plots in Figure 3 across the line $w^{1}=w^{2}$. For the corticostriatal model, this swaps between equilibria at $\left(w^{1*},w^{2*}\right)$ and $\left(w^{2*},w^{1*}\right)$. After a swap the old equilibrium is no longer a zero of the dynamical system, and because the weights are generally not too close to 0 when α is not too large, the drift rate will not be very small. Thus, the weights can quickly move to the new equilibrium. For the additive model, the weights tend to converge closer to 0 during the learning process. Since the weight drift equation (12) is proportional to w, this can cause the weights to become stuck close to the origin. The weight drift is also proportional to E[p](1-E[p]), and since the action selection probability is typically higher for the additive model than for the corticostriatal model after convergence (because the difference between the weights $w^{1}-w^{2}$ is typically greater), this term will be smaller for the additive model than for the corticostriatal model. These factors lead to slower and less consistent switching. For the symmetric model, the weight drift equation is proportional to $w^{2}(1-w)$. This term severely slows down changes in weights near both 0 and 1, leading to very slow switching. Running the simulations with longer intervals between switches would generally not help as the weights take just as much time to escape from these values as they spend approaching them; that is, longer intervals lead to stronger convergence and hence more time needed to move away after a contingency switch.

**Fig. 6:**
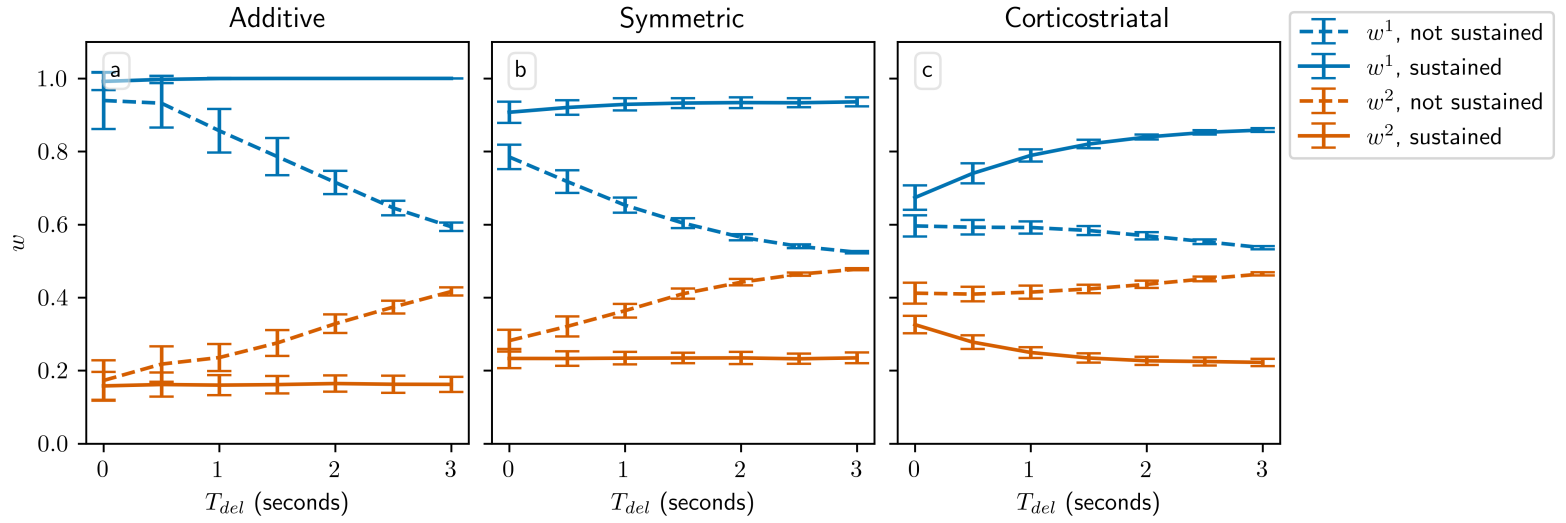
Performance in the action selection setting as delay is varied with and without sustained activity. Plots show weights after 1000 steps for additive (a), symmetric (b), and corticostriatal (c) models. With no sustained activity, both input channels are silenced during the delay period, while with sustained activity, the input to the selected channel is maintained at a level of $a_{\text{sel}}$ (see Figure 2)

All of our analytical results in this setting depend on a key assumption: that the delay $T_{\text{del}}$ is long relative to the eligibility timescale $\tau_{\text{eli}}$. By imposing a large gap between when the firing rate is measured, using equation (6), and when dopamine is actually released, the delay ensures that the dopamine signal is statistically independent of the other terms in the weight update equation. This independence is what allows us to factor out the dopamine term in the average drift formulas, equations (12) and (14). Without this delay, correlations between the dopamine and the eligibility prevent us from deriving a corresponding averaged model. To judge the generalizability of our analytical results, we test model performance as $T_{\text{del}}$ is varied using simulations. Figure 6 shows the weight distributions after 1000 steps for the three models as a function of $T_{\text{del}}$. We also compare to an alternative task implementation without sustained activity in the selected channel. We can see that when activity is sustained in the selected channel, reducing $T_{\text{del}}$ either does not meaningfully alter performance, in the case of the additive and symmetric models, or degrades performance somewhat by decreasing the difference between the weights $w^{1}-w^{2}$, in the case of the corticostriatal model. In contrast, without sustained activity, learning is only possible when $T_{\text{del}}$ is sufficiently small. In this setting, the only way for the system to learn is for eligibility from spikes within the spike count window to persist until dopamine is released. As $T_{\text{del}}$ gets larger, the rate of learning decreases due to the exponential decay of the eligibility. Thus we see that using sustained activity in the selected channel effectively solves the credit assignment problem [35, 44] and results in a system that performs well when the delay is large, while without it the system is only able to successfully produce large differences between $w_{1}$ and $w_{2}$, and hence learn the task, when $T_{\text{del}}$ is small.

Overall, while the additive and symmetric models do well in the action selection setting when reward contingencies are static, the corticostriatal model does much better when they can change over time. The additive and symmetric models also seem more sensitive to α, with their performance tending to degrade when α is too large. The weakness of the corticostriatal model is that the weights tend to remain in a somewhat narrow band compared to the other two models (which can, if given enough time, drive the weights arbitrarily close to 0 and 1), leading to a lower probability of taking the correct action. This probability is determined by the number of postsynaptic spikes in each channel, and if the weights are close together, then spiking noise will sometimes lead to more spikes being counted in the incorrect channel, causing the wrong action to be taken. This outcome occurs despite the fact that here we use a large value of β, the temperature parameter in our action selection probability function. We believe that this problem is not a fundamental one, however, as it can be easily solved through downstream integration over the outputs of multiple striatal neurons to obtain a clearer signal. Consequently, we conclude that overall, the corticostriatal model is better suited to the action selection setting than are the additive and symmetric models.

### 3.2 Value Estimation Setting

#### 3.2.1 Analysis of Dynamics

The strong performance of the corticostriatal model in the action selection setting with contingency switches highlights its flexibility and raises the question of whether it is equally suited to learn to estimate choice values, or whether other plasticity rules will perform better for such tasks. We attempt to answer this question using the value estimation task described in Section 2.2. Under suitable assumptions it is possible to derive a formula for the average drift of the weights and action probabilities over time for the additive and symmetric models in this setting:

(15) $${\dot{w}}_{i}=\left(pR_{1}^{*}+(1-p)R_{2}^{*}-\frac{\left\langle w,r\right\rangle }{N}\right)r^{\text{dop}}\tau_{\text{dop}}\tau_{\text{eli}}\frac{\lambda }{N}\left(\tau \langle w,r\rangle \Delta f\left(w_{i}\right)r_{i}+f_{+}\left(w_{i}\right)w_{i}r_{i}\right),$$

(16) $$\dot{p}=\overset{‾}{\lambda }\beta r^{\text{dop}}\tau_{\text{dop}}p(1-p)\left(p\left(R_{1}^{*}-\frac{\left\langle w,r\right\rangle }{N}\right)-(1-p)\left(R_{2}^{*}-\frac{\left\langle w,r\right\rangle }{N}\right)\right),$$

where $\Delta f=f_{+}-f_{-}$ and p is the probability of selecting action $A_{1}$. See Appendix A for the derivation of system (15,16). As in the action selection setting, we assume that the delay $T_{\text{del}}$ is large relative to $\tau_{\text{eli}}$.

The terms in equations (15) and (16) have simple interpretations. In equation (15), the $\tau \langle w,r\rangle \Delta f\left(w_{i}\right)r_{i}$ and $f_{+}\left(w_{i}\right)w_{i}r_{i}$ terms are identical to those in the action selection setting and correspond to independent spike pairs and pre-post pairs where the presynaptic spike causes the postsynaptic spike, respectively. The $pR_{1}^{*}+(1-p)R_{2}^{*}-\langle w,r\rangle /N$ term is the average dopamine level, which is the difference between the average reward received and the average output firing rate. In equation (16), p(1-p) arises from the logistic dependence on ${\overset{‾}{R}}_{1}-{\overset{‾}{R}}_{2}$ in equation (7), and the other term describes the expected difference in dopamine levels between trials where action $A_{1}$ is selected and those where $A_{2}$ is selected.

System (15,16) has a number of equilibria. The following are critical points for every choice of $f_{+},f_{-}$:

$$p=0,\frac{\left\langle w,r\right\rangle }{N}=R_{2}^{*},$$

$$p=1,\frac{\left\langle w,r\right\rangle }{N}=R_{1}^{*},$$

$$p=\frac{R_{2}^{*}}{R_{1}^{*}+R_{2}^{*}},0,\;\text{or}\;1,w=0.$$

Note that the first two families of equilibria comprise (N-1)-dimensional hyperplanes of weights at fixed p. These correspond to sets of weights such that the average output firing rate ⟨w,r⟩/N equals the average reward received. (In general, for other values of p, the set of weights where $\langle w,r\rangle /N=pR_{1}^{*}+(1-p)R_{2}^{*}$ will have zero weight drift, $\dot{w}=0$, but $\dot{p}$ may not be zero.) Different choices of $f_{+},f_{-}$ may lead to additional equilibria. In particular, the symmetric model has a set of extra critical points at:

$$p=\frac{R_{2}^{*}-\|r{\|}_{1}/N}{R_{1}^{*}+R_{2}^{*}-2\|r{\|}_{1}/N},0,\;\text{or}\;1,w=1.$$

It also has a number of other equilibria which appear when N>1, which we describe in Appendix B.3.

Assuming $R_{1}^{*}>R_{2}^{*}$, so that action $A_{1}$ is more rewarding than $A_{2}$, we would like w and p to converge to the $p=1,\langle w,r\rangle /N=R_{1}^{*}$ set of equilibria, at which the agent always picks $A_{1}$ and the system accurately predicts the reward. Conversely, if $R_{1}^{*}<R_{2}^{*}$ we would like the system to converge to the $p=0,\langle w,r\rangle /N=R_{2}^{*}$ set of equilibria. In Appendix B.1 we prove the following result providing a condition relevant to the stability of these states:

**Theorem 2** *Pick* w *and* p *so that either* $p=1,\langle w,r\rangle /N=R_{1}^{*}$ *or* $p=0,\langle w,r\rangle /N=R_{2}^{*}$. *For the additive and symmetric models given by* equations (A9) *and* (16), *the Jacobian at this equilibrium has one nonzero eigenvalue, which has an eigenvector that is zero in the* p *coordinate. This eigenvalue is negative if and only if*

(17) $$0<\sum_{i=1}^{N}r_{i}^{2}\left(\tau NR^{*}\Delta f\left(w_{i}\right)+f_{+}\left(w_{i}\right)w_{i}\right),$$

*with the choice of* $R^{*}=R_{1}^{*}$ *or* $R^{*}=R_{2}^{*}$ *corresponding to the equilibrium under examination*.

**Fig. 7:**
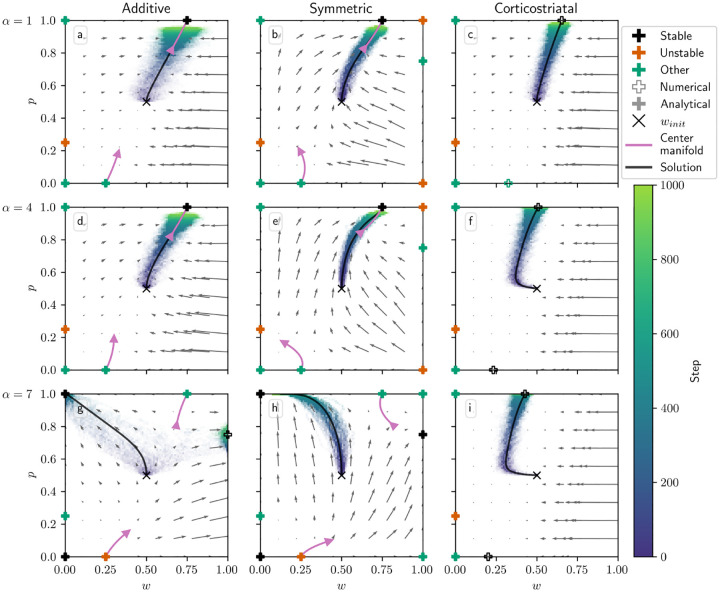
Distribution of w and p over time in the value estimation setting as α is varied. Columns show the additive (a, d, g), symmetric (b, e, h), and corticostriatal (c, f, i) models. α is varied across rows: (a-c) α=1; (d-f) α=4; (g-i) α=7. We use $w_{\text{init}}=0.5,p_{\text{init}}=0.5$ here, marked by the “×“ in each plot. The low-order shape of the center manifolds for the additive and symmetric models at certain critical points is plotted in pink. The numerical solution to the averaged system starting at $w_{\text{init}}=0.5,p_{\text{init}}=0.5$ is plotted in gray and closely aligns with the center manifold in (a,b,d,e)

At these equilibria there also exists a center manifold with nonzero component in the p coordinate. The union of these manifolds over the equilbrium hyperplanes forms an N-dimensional surface, which in many cases appears to connect the two sets of equilibria at p=0 and p=1 (see Figure 7). We show in Appendix B.1 that the flow along this manifold depends on the sign of $R_{1}^{*}-R_{2}^{*}$:

**Theorem 3** *Points of the form* $p=0,\langle w,r\rangle /N=R_{2}^{*}$ *or* $p=1,\langle w,r\rangle /N=R_{1}^{*}$ *are equilibria of* equations (A9) *and* (16) *with a nontrivial center manifold; the flow along this manifold is towards* p=1 *and away from* p=0 *if* $R_{1}^{*}>R_{2}^{*}$, *or away from* p=1 *and towards* p=0 *if* $R_{1}^{*}<R_{2}^{*}$.

Thus, as long as equation (17) is satisfied for a sufficient proportion of the hyperplane, the system will generally move along the center manifold towards the appropriate set of equilibria, where the more rewarding action is always picked and the reward is predicted accurately. The presence of other equilibria may complicate this picture in some cases, though. In Table 3 we summarize the equilibria of the additive and symmetric models and the conditions determining their stability; see Appendix B for proofs of all of these results. When N>1, the symmetric model has a number of additional equilibria not included in the table that we do not analyze in detail; see Appendix B.3.

The corticostriatal model is more difficult to analyze. Because the form of the plasticity rule in that model depends on the sign of the dopamine signal, in general it is not possible to factor out the average dopamine level $pR_{1}^{*}+(1-p)R_{2}^{*}-\langle w,r\rangle /N$ like we can for the additive and symmetric models. We analyze this model further in Appendix A.2 and Appendix B.4; its equation for the dynamics of p is identical to equation (16), but that for w differs from equation (15). In general, points on the hyperplanes $\langle w,r\rangle /N=R_{1}^{*}$ or $R_{2}^{*}$ will not be critical points under this model, meaning that the weights will generally not converge to the optimal values.

#### 3.2.2 Simulation Results

Figure 7 illustrates the evolution of (w,p) with N=1 for all three plasticity models, overlaid on the phase portraits for the corresponding averaged models, for three different values of α. Here we plot w along the x-axis and p along the y-axis. In addition to the averaged model vector fields, we also include a representation of the lower-order behavior of the center manifolds described above.

We can see several of the changes in behavior described in Table 3 as α increases. For the additive model, when α is not too large, p converges to 1 and w converges to $R_{1}^{*}/r$, so that the predicted reward equals $R_{1}^{*}$. (Recall that $R_{1}^{*}>R_{2}^{*}$, so that action $A_{1}$ is more rewarding.) However, for large enough α (in this case α≥6) the condition given in equation (17) fails, and the equilibrium loses stability. For the additive model in the N=1 case this condition is equivalent to $\tau (\alpha -1)<1/\|r{\|}_{1}$, so as described in Table 3, the three equilibria along w=0 also change their stability as α increases. For the initial conditions and parameters we used, this leads to most trajectories converging to p=1,w=0 for α=7.

Note that an extra critical point arises in the α=7 plot at p≈0.75,w=1. Like we saw in the action selection setting, this is not a “true” critical point but rather the result of the weights being clipped at 0 and 1. In this case, it can be seen by examining equation (16) that at w=1 and $p=\left(R_{2}^{*}-\|r{\|}_{1}/N\right)/\left(R_{1}^{*}+R_{2}^{*}-2\|r{\|}_{1}/N\right)=0.75$, using the parameters listed in Table 2, we have $\dot{p}=0$ and $\dot{w}>0$, so preventing the weights from increasing above 1 makes trajectories accumulate at this point.

The symmetric model behaves very similarly to the additive model, except that it has three extra equilibria at w=1. For low values of α, these are unstable or saddles, and the flow is away from w=1. Past α=6 when the $\tau (\alpha -1)<1/\|r{\|}_{1}$ condition fails, w=1 becomes attractive, and in particular the fixed point at $p=\left(R_{2}^{*}-\|r{\|}_{1}/N\right)/\left(R_{1}^{*}+R_{2}^{*}-2\|r{\|}_{1}/N\right),w=1$ becomes stable. While in the simulation shown in Figure 7h trajectories generally do not converge to it, the vector field indicates that under different initial conditions trajectories will converge to it rather than to the p=1,w=0 equilibrium.

For the corticostriatal model, while we are able to analytically determine the stability of the critical points along the w=0 axis (see Appendix B.4), we are unable to find a closed-form expression for the two critical points evident in each of Figure 7c,f,i for nonzero values of w; these are instead computed numerically. Note that these *are* zeros of the underlying dynamical equations, unlike the extra equilibria we see in the additive model; they simply cannot be expressed analytically. Both the upper and lower critical points appear to remain stable as α increases, although their position shifts with α. In general, they will not equal $w=R_{1}^{*}/r$ or $w=R_{2}^{*}/r$, so while the system may learn to pick the more rewarding action (based on p→1), the value estimates made by the system will be incorrect.

For the additive and symmetric models, we also plotted curves to indicate the low-order shape of the center manifolds at the $p=0,w=R_{2}^{*}/r$ and $p=1,w=R_{1}^{*}/r$ critical points (Figure 7, purple curves). As can be seen from comparison with the vector field, especially in the symmetric model, the curves describe the local behavior of trajectories near these equilibria. Note that the lengths of these curves were selected for visualization of the manifolds’ shapes and should not be taken to indicate the regions where this low-order approximation holds; for example, for α=7, the vector field and center manifold align only on a very small neighborhood of the p=1 equilibria.

**Fig. 8:**
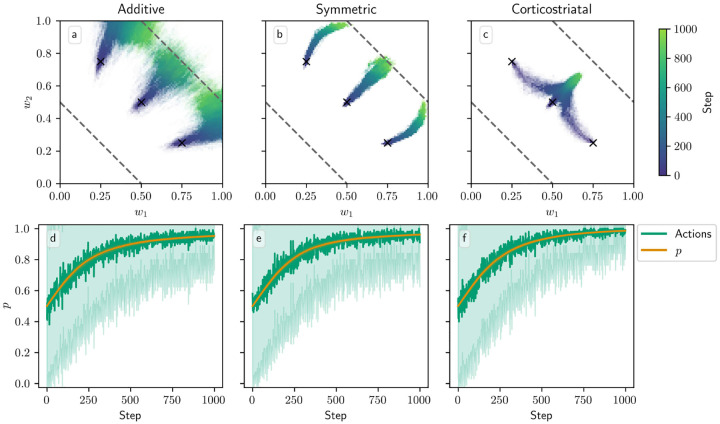
Distribution of w and p over time in the value estimation setting with N=2. The color code indicated in the color bar shows the simulation step. Columns show the additive (a, d), symmetric (b, e), and corticostriatal (c, f) models. The top row shows the two weights, $w_{1}$ and $w_{2}$, while the bottom row shows the proportion of trials on which action $A_{1}$ is picked along with the values of p. The two sets of equilibria at $\langle w,r\rangle /N=R_{1}^{*}$ and $R_{2}^{*}$ are indicated with dashed lines. (Note that these are not equilibria for the corticostriatal model; we plot the lines there only for ease of comparison.) We use α=1 and three different initial conditions: $w_{\text{init}}=(0.25,0.75)$, $w_{\text{init}}=(0.5,0.5)$, and $w_{\text{init}}=(0.75,0.25)$ (all with $p_{\text{init}}=0.5$), indicated by the “×“ marks in the upper plots; the lower plots only include p values and action proportions for $w_{\text{init}}=(0.5,0.5)$ as the other initial conditions give almost identical results. In the lower plots, the shaded envelopes show standard deviations while solid lines show means over 100 trials

In Figure 8, we plot the dynamics when N=2, starting from three different initial conditions to better illustrate the dynamics. In this case the sets of equilibria $\langle w,r\rangle /N=R_{1}^{*}$ and $R_{2}^{*}$ form lines in the $w_{1}-w_{2}$ plane, indicated in the figure with dashed lines. We can see that for the additive and symmetric models, the weights converge to the $\langle w,r\rangle /N=R_{1}^{*}$ line as p converges to 1, indicating that the more rewarding action is being picked more often, and that the system correctly estimates the values of its rewards. There does appear to be some diffusion along this line, especially for the additive model. For the corticostriatal model, while p converges to 1, the weights appear to converge to a point somewhat off of the line; as we saw in the N=1 case, the value estimates made by the system will be incorrect.

**Fig. 9:**
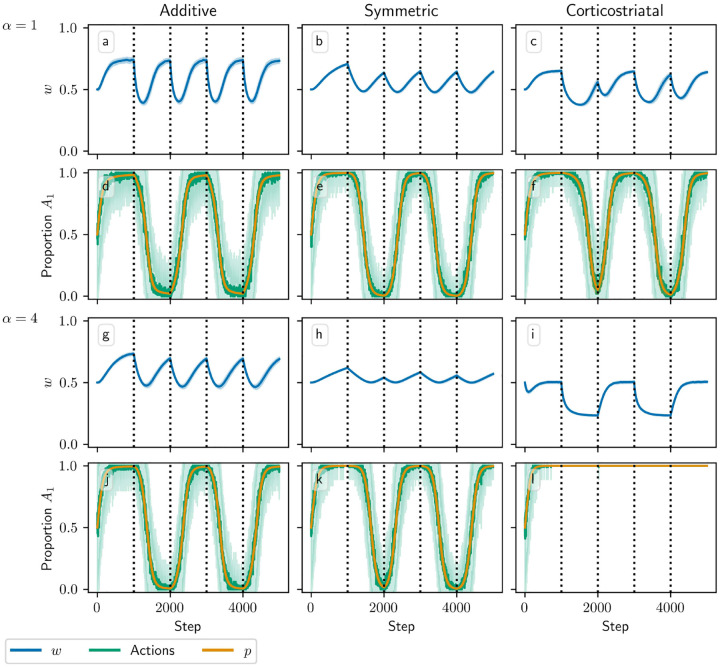
Model performance in the value estimation setting with contingency switching as α is varied. Plots show weights (a-c, g-i) and the proportion of trials on which action $A_{1}$ is picked along with the values of p (d-f, j-l) versus time for the additive (a, d, g, j), symmetric (b, e, h, k), and corticostriatal (c, f, i, l) models. α is varied across rows: (a-f) α=1; (g-l) α=4. Here reward contingencies switch between $R_{1}^{*}=7.5,R_{2}^{*}=2.5$ and $R_{1}^{*}=2.5,R_{2}^{*}=7.5$ every 1000 steps; we use λ=0.00015 and $\overset{‾}{\lambda }=0.005$ for illustrative purposes

In Figure 9 we test a version of the value estimation task with contingency switching, in which we switch which action leads to which reward every 1000 steps. In general, performance with contingency switching depends on the balance between the learning rates for w and p,λ and $\overset{‾}{\lambda }$. The learning rates need to be large enough that both w and p have enough time between contingency switches to approach close to their equilibrium values, but ideally not so large that the system is too noisy. Additionally, performance is significantly degraded when λ is large relative to $\overset{‾}{\lambda }$. For instance, suppose $R_{1}^{*}>R_{2}^{*}$, and we switch from a state in which action $A_{1}$ leads to reward $R_{1}^{*}$ and $A_{2}$ leads to $R_{2}^{*}$ to one where $A_{1}$ leads to $R_{2}^{*}$ and $A_{2}$ leads to $R_{1}^{*}$. In the original state, $A_{1}$ is more highly rewarded, so suppose p≈1 and $\langle w,r\rangle /N\approx R_{1}^{*}$, matching the convergence behavior of the additive and symmetric models. If λ is too large relative to $\overset{‾}{\lambda }$, then after the switch, w may rapidly converge to $\langle w,r\rangle /N\approx R_{2}^{*}$ while p remains close to 1. Since the system is now accurately predicting the low reward it is receiving, the dopamine signal will be small, and so p will take a long time to leave the vicinity of 1. For this reason the system generally performs better with contingency switching when $\overset{‾}{\lambda }$ is large relative to λ.

Another potential issue occurs if the weights do not approach $\langle w,r\rangle /N\approx R_{1}^{*}$ or $R_{2}^{*}$ closely enough between contingency switches. This pitfall always arises with the corticostriatal model, as these hyperplanes are not equilibria for this model, but it also may occur in other cases if λ is too small. If the weights do not converge properly between switches, then p may converge to 0 or 1 *too strongly*. To see this, observe that near p=0 or $p=1,\dot{p}$ is proportional to the estimated value term $R_{2}^{*}-\langle w,r\rangle /N$ or $R_{1}^{*}-\langle w,r\rangle /N$, respectively (see equation (16)). If w is close to its equilibrium value, then this term will be small, and generally p will not approach 0 or 1 too closely. However, if it does not converge properly, the estimated value term will remain large and p will be driven to 0 or 1 exponentially, as $\dot{p}$ will be proportional to p(1-p) times a factor significantly different from zero. This is problematic in contingency switching tasks because it may then take too long for p to leave this point when contingencies swap.

Due to these considerations, each of the three plasticity rules needs different values of λ and $\overset{‾}{\lambda }$ to perform well. In Figure 9 we use λ=0.00015 and $\overset{‾}{\lambda }=0.005$, in which all three models do at least moderately well at selecting the correct action when α=1, although the symmetric and corticostriatal models do not do as well at accurately predicting their rewards. A higher λ would help the symmetric model but harm the corticostriatal model, which would benefit from a lower λ, and all three models require a higher $\overset{‾}{\lambda }$ than the value of 0.0025 used in other figures. Performance of these models also degrades significantly for larger values of α, although different choices for λ and $\overset{‾}{\lambda }$ may ameliorate this.

**Fig. 10:**
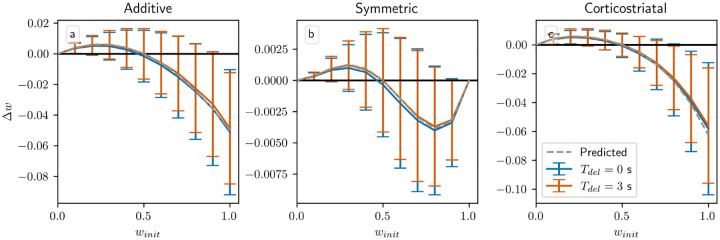
Weight drift after a single dopamine release in the value estimation setting with variable $T_{\text{del}}$. Plots show results for the additive (a), symmetric (b), and corticostriatal (c) models, with $T_{\text{del}}=0\;\text{s}$ and $T_{\text{del}}=3\;\text{s}$, as well as the predicted weight drift based on the averaged models, as $w_{\text{init}}$ is varied for N=1, with $p_{\text{init}}=0.5$ fixed. Note that when $w_{\text{init}}=1$ there are some deviations from predictions even for $T_{\text{del}}=3\;\text{s}$ due to boundary effects not taken into account by the averaged models. Error bars show standard deviations over 10000 samples

All of our analytical results in this setting depend on the assumption that the delay $T_{\text{del}}$ is long relative to $\tau_{\text{eli}}$, like in the action selection setting. This assumption is what allows us to factor out the dopamine term $pR_{1}^{*}+(1-p)R_{2}^{*}-\langle w,r\rangle /N$ in the average drift formula, equation (15). Without this term, we can no longer guarantee that points on the hyperplanes $\langle w,r\rangle /N=R_{1}^{*}$ or $R_{2}^{*}$ are equilibria for any of the three plasticity models. In Figure 10 we plot the change in weight after a single dopamine release as a function of $w_{\text{init}}$ in an N=1 setting, with $p_{\text{init}}=0.5$ fixed, while varying $T_{\text{del}}$. When $T_{\text{del}}=3\;\text{s}$ the simulations obey the predictions of the averaged models, and in the additive and symmetric cases they intersect the x-axis at w≈0.5, the point at which $wr=pR_{1}^{*}+(1-p)R_{2}^{*}$ when p≈0.5. (While the plots may appear fairly noisy, keep in mind that they only display the change in weight after a single dopamine signal. Figure 7 shows that although there is some dispersion, over the course of many trials trajectories still tend to follow the averaged dynamics.) When $T_{\text{del}}=0\;\text{s}$ the simulations do not exactly match the predictions, but the differences are fairly small; they are most visible for the symmetric model. In most cases where they differ the $T_{\text{del}}=0\;\text{s}$ curves are below the curves for $T_{\text{del}}=3\;\text{s}$. This undershoot may occur because there is a source of negative correlation between the dopamine value and the eligibility at the time that dopamine is released. Specifically, the dopamine value D from equation (10) is negatively correlated with the number of postsynaptic spikes in the spike count window, while the eligibility will in most cases (depending on the plasticity model and the parameters) be positively correlated with the number of recent spikes. If there is no delay, then this will include the spikes in the spike count window used to compute the dopamine value. While these plots show that for realistic parameter values our model is not very sensitive to the delay or its absence, it should be noted that for other sets of parameters, for instance smaller values of $\tau_{\text{dop}},\tau_{\text{eli}}$, and $T_{\text{win}}$, the lack of a delay can have a significant effect.

**Fig. 11:**
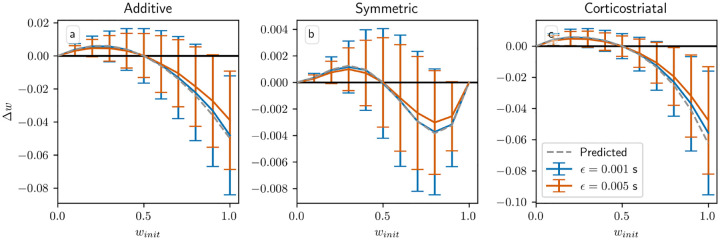
Weight drift after a single dopamine release in the value estimation setting across two choices of ϵ. Plots show results for the additive (a), symmetric (b), and corticostriatal (c) models, with ϵ=0.001s and ϵ=0.005s, as well as the predicted weight drift based on the averaged models, as $w_{\text{init}}$ is varied for N=1, with $p_{\text{init}}=0.5$ fixed. Note that larger ϵ generally leads to less variability in Δw and that when $w_{\text{init}}=1$ there are some deviations from predictions even for ϵ=0.001s due to boundary effects not taken into account by the averaged models. Error bars show standard deviations over 10000 samples

Another assumption in our analysis is that ϵ, the time between presynaptic spikes and any postsynaptic spikes they cause, is small relative to τ, the time constant of synaptic plasticity. Specifically, we assume following [24] that $e^{-\epsilon /\tau }\approx 1$; using our default values of ϵ=0.001s and τ=0.02s, this quantity is $e^{-\epsilon /\tau }=0.95$. In Figure 11 we show the result of increasing ϵ to 0.005 s, in which case $e^{-\epsilon /\tau }=0.78$. The main effect of increasing ϵ is to reduce the magnitude of the changes in weight. τ defines the duration of the window of synaptic plasticity; as ϵ increases, pre- and postsynaptic spikes grow farther apart relative to τ, and so weight changes due to presynaptic spikes directly causing postsynaptic spikes, corresponding to the $f_{+}\left(w_{i}\right)w_{i}r_{i}$ term in equation (15) for the additive and symmetric models (the other terms correspond to spike pairs that are close together only by chance), are reduced by a factor of $e^{-\epsilon /\tau }$. Overall, though, for realistic values of ϵ the differences between the two curves are small, and the qualitative behavior is largely independent of ϵ.

## 4 Discussion

Accurately modeling learning in the cortico-basal ganglia-thalamic circuit requires the use of an appropriate synaptic weight update rule for the dopamine-dependent STDP in the corticostriatal connections. In this paper we examine three plasticity models that combine dopamine, eligibility, and spike timing signals in different ways – the additive, symmetric, and corticostriatal models – and evaluate their performance in various task settings. We find that the corticostriatal model is able to rapidly relearn swapped reward contingencies in the action selection setting. This rapid learning matches the results seen in experiments with animals [49] and humans [50]. However, the corticostriatal model performs poorly in the value estimation setting, as the synaptic weights do not converge to values that allow the agent to accurately estimate the rewards it will receive. In contrast, the additive and symmetric models are able to learn to accurately estimate reward values. But while they can accomplish a simple action selection task, they – especially the symmetric model – do not perform as well when reward contingencies occasionally switch, because their weights tend to become stuck at or near the boundaries of the domain. They are also more sensitive to α than the corticostriatal model in this setting, with larger values degrading their performance significantly. Overall, we find that the choice of which plasticity model to use can have a large impact on the dynamics of synaptic weights and hence on both the learning achieved by the circuit and the ability of the model to perform a given task. Which plasticity model is appropriate depends strongly on the tasks it will be asked to perform. Ultimately, these results suggest that different synaptic plasticity mechanisms may be at play at corticostriatal synapses involving different regions of the striatum with distinct functions, as well as at corticocortical synapses with dopamine-dependent plasticity [51], and that additional experimental and theoretical work is needed to pin down the precise forms of plasticity that occur at corticostriatal synapses and how they should be modeled.

Our mathematical analysis shows how the choice of parameter values impacts model performance on the tasks we considered. Specifically, we characterized how the stability of various equilibria (and therefore the ability of the models to solve each task) depends on the parameters $a_{\text{sel}}$ (for the action selection setting), $\alpha ,\tau ,r,R_{1}^{*}$, and $R_{2}^{*}$. The parameters α, which represents the weighting of negative eligibility relative to positive in the additive and symmetric models and the weighting of weight decreases relative to increases in the corticostriatal model, and τ, the STDP time constant, have a strong impact on model performance In general, increasing α or τ will reduce the ranges of input firing rates r and rewards $R_{1}^{*},R_{2}^{*}$ for which the system converges to the desired equilibria. Therefore, these parameters are particularly important for practitioners to understand and to select judiciously.

Why exactly do the three plasticity models run into difficulties in some settings? As discussed in Section 2.1.2, an important issue with the additive model is that it does not prevent weights from being driven to the boundaries (or past them, if the weights are not artificially cut off). The symmetric and corticostriatal models represent two alternative approaches to resolving this issue. (The multiplicative model used in prior work does not solve the issue when modified to incorporate dopamine.) The symmetric model keeps the weights bounded by multiplying the weight update equations by w(1-w). However, in some cases this leads to the weights becoming stuck near the domain boundaries, resulting in poor performance in the action selection setting with contingency switching (Figure 5). The corticostriatal model, on the other hand, keeps the weights bounded by using scaling factors w or 1-w depending on the sign of the product of the dopamine signal term with the eligibility trace term. In other words, it ensures that a scaling factor w is used when weights decrease and 1-w when weights increase (see Table 1). The resulting model exhibits very good performance in the action selection setting with contingency switching. The cost of this plasticity rule, from an analytical perspective, is that the dopamine signal can no longer be factored out of the weight drift equation in the value estimation setting. Consequently the corticostriatal model features nonzero mean weight drift even when the mean dopamine signal is zero, leading to its failure to converge to the equilibria representing accurate value estimates in the value estimation setting.

One important issue that we have highlighted throughout this work is the impact that delays have on the weight dynamics. In the action selection setting, we saw that in order for effective learning to take place when there is a substantial delay between when we estimate the postsynaptic firing rate and when dopamine is actually delivered, we need to sustain activity in the selected channel. Without this sustained activity, performance quickly degrades as the delay increases (see Figure 6). With sustained activity, though, performance is maintained or actually improves as the delay increases. Likewise, in the value estimation setting, we require a long delay to ensure convergence to the equilibria representing accurate value estimation. Without this delay, we cannot guarantee convergence to the solution plane due to correlations between terms (although in practice this usually does not substantially affect the results). A complicating factor that we have not addressed is that experimental results consistently show that dopamine release immediately upon pre-post spike pairing does *not* lead to a change in weight; rather, the dopamine must come some time after the spiking activity to effect significant synaptic changes [34, 47]. Moreover, dopamine is not released instantly, but rather takes some time to ramp up to its peak value [46]. These findings raise important questions about how to best understand and model delays within a synaptic plasticity framework. Although we considered both the dopamine concentration and the eligibility trace as jumping up immediately and then decaying exponentially, for the sake of analytical tractability and for consistency with prior computational work, an important extension of these results would be to represent these signals as slowly ramping up and then ramping down over time and to study how these more realistic time-courses interact with delays and what computational outcomes they produce.

Our results build on past literature on spike-timing dependent and corticostriatal synaptic plasticity. Our additive model is based on the plasticity rules described in [24], but our plasticity rule differs from theirs in that we incorporate dopaminergic modulation of the synaptic plasticity. The settings in which we evaluate the model also differ significantly from theirs: while they study conditions under which symmetry breaking in the weight distributions occurs and when the models can learn to represent correlations in a set of inputs, we focus on measuring performance in more complex settings associated with the functional roles of corticostriatal synapses within cortico-basal ganglia-thalamic circuits. The corticostriatal model in this paper is based on the plasticity model used in [21] but differs from their model in a number of important ways. We make several simplifications to the model, including setting the scaling factors and time constants for pre- and postsynaptic spike traces equal to each other (in their notation, $\tau_{PRE}=\tau_{\text{POST}}$ and $\Delta_{PRE}=\Delta_{\text{POST}}=1$), as well as considering a single class of striatal neurons rather than taking into account the existence of multiple striatal neuron subpopulations with different plasticity properties. They also employ their plasticity model in a more biologically realistic setting, incorporating many components of the basal ganglia circuitry that we leave out. A conceptually interesting difference between our models is that they use a single eligibility trace, summing up both positive (corresponding to pre-before-post spike pairs) and negative (post-before-pre) contributions, while we use two different traces for the positive and negative components. The use of two traces is justified by experimental evidence suggesting that the brain uses two distinct eligibility traces for LTP and LTD [37]. Note, however, that the computational model introduced in [37] differs considerably from the models used here, as it does not use αw or 1-w factors to rescale the positive and negative traces, instead simply adding them together without modification. We find in Appendix D that altering our models to employ a single eligibility trace leads to qualitatively similar results in most cases, although the single-trace models are much more difficult to analyze mathematically.

In both the action selection and value estimation settings that we have considered, rewards are deterministic, with the agent always receiving reward $R_{1}^{*}$ after taking action $A_{1}$ and $R_{2}^{*}$ after taking $A_{2}$. One could also consider versions of these settings with probabilistic rewards, where taking action $A_{1}$ leads to reward $R_{1}^{*}$ with some fixed probability q and to $R_{2}^{*}$ with probability 1-q, and vice versa for action $A_{2}$. In the value estimation setting, where we are able to calculate explicit formulas for the average dynamics (see Appendix A and the discussion in Section 3.2), it is easy to see that our results can accommodate probabilistic rewards by simply replacing $R_{1}^{*}$ and $R_{2}^{*}$ with $qR_{1}^{*}+(1-q)R_{2}^{*}$ and $(1-q)R_{1}^{*}+qR_{2}^{*}$, respectively, in the equations for the average dynamics (equations (15) and (16)). In the action selection setting the expressions for $E\left[R^{*}\right]$ and E[p] in equation (8) would need to be modified to properly compute the expected reward when rewards are probabilistic, but otherwise the qualitative behavior will be similar to the case of deterministic rewards.

Another direction for future work would be to extend our models to more complex settings in which both action probability learning and value learning are modeled in a biologically detailed way involving synaptic plasticity; possibly, thalamostriatal inputs, which we have ignored but have been considered elsewhere, could be important in this process as well [52, 53]. A number of other three-factor plasticity rules, which we did not consider, have been explored in the literature. One important model can be found in [54]; while our learning rules are generally built from two-factor rules modified to incorporate dopaminergic feedback, they derive their learning rule directly from gradient ascent applied to a reward signal. Another work modeling dopamine-dependent STDP is [55]. The plasticity rule in that work closely resembles our additive model. However, while we focus on the corticostriatal synapses and employ a simple setting consisting of a population of cortical neurons connected to a single striatal neuron, they instead use a mixed population of excitatory and inhibitory neurons with random connectivity meant to model part of a cortical column. The scenarios that they use to test their model also differ from ours. For a more detailed review of other work on three-factor plasticity rules, see [3, 4].

In addition to considering more complicated task settings, future work could include additional biological realism into our modeling framework. It is certainly possible that the plasticity mechanism used in the corticostriatal synapses incorporates features that are not well-captured by any of the models considered here. It is also possible that the simplified models that we consider omit aspects of the computational structure of the basal ganglia that are crucial for functional performance. For instance, we do not model the competition between direct and indirect pathways through the basal ganglia, nor the differing effects of dopamine on the two pathways [29, 32]. There may also be more complexity to dopaminergic feedback than the simple model we use; for example, recent work suggests that the dopamine signal may be better modeled as multidimensional rather than scalar-valued [56]. An exciting future direction would be to extend our analysis to take more of these subtleties into account. Nevertheless, we believe that the settings that we studied capture key computational components that will remain relevant in more detailed models and hence that our results will be informative in such settings.

Overall, what are the implications of our findings for models of the basal ganglia? We showed that each model does well in some settings yet fails to accomplish the given task in others. An interesting possible implication of our work is that different regions of the striatum may feature different plasticity mechanisms specialized to their particular roles. For instance, the ventral and dorsal striatum, which primarily contribute to value estimation and action selection, respectively [57], may use distinct plasticity rules tuned to the specific tasks that they perform, as suggested by experimental evidence [15, 17]. More generally, while we have focused in this paper on the corticostriatal connections, our framework is general enough that it may apply to any other region of the brain that receives dopaminergic signals, such as the prefrontal cortex, where dopamine-dependent plasticity also occurs [51]. The task settings we considered represent fairly general models of different aspects of the learning dynamics under dopaminergic feedback that do not depend heavily on the architecture of the basal ganglia. Thus, the fact that no plasticity rule performed well in every setting in our study supports the general principle that different brain regions should exhibit features – in this case, synaptic plasticity mechanisms – that are specialized for the specific computational functions that they perform.

## Supplementary Material

1

## Figures and Tables

**Table 1:** Scaling factors for the plasticity rules for positive and negative dopamine and eligibility

	Additive		Multiplicative		Symmetric		Corticostriatal	
_$E_{i}(t)$_╲^D(t)^	−	+	−	+	−	+	−	+
−	α	α	αw	αw	αw(1-w)	αw(1-w)	1-w	αw
+	1	1	1-w	1-w	w(1-w)	w(1-w)	αw	1-w

Blue cells correspond to scenarios in which the weights will increase, while orange indicates that the weights will decrease

**Table 2:** Default simulation parameters for the two main task settings

	Action Selection	Value Estimation
Samples	100	100
Steps	1000	1000
λ	0.01	0.001
$\overset{‾}{\lambda }$	N/A	0.0025
$w_{\text{init}}$	0.5	0.5
$p_{\text{init}}$	N/A	0.5
N	1 or 2	1 or 2
r	10 or (15, 5) s^−1^	10 or (10, 10)s^−1^
$R_{1}^{*},R_{2}^{*}$	(2,1)	(7.5, 2.5)
α	1	1
τ	0.02 s	0.02 s
$\tau_{\text{dop}}$	1 s	1 s
$\tau_{\text{eli}}$	1 s	1 s
$T_{\text{del}}$	10 s	3 s
$T_{\text{win}}$	1 s	1 s
ϵ	0.001 s	0.001 s
$r^{\text{dop}}$	1/21 s^−1^	1/7 s^−1^
β	10^6^	1
$a_{\text{sel}}$	0.7	N/A

**Table 3:** Equilibria of the additive and symmetric models in the value estimation setting

Equilibria	Dynamics
$p=0,\langle w,r\rangle /N=R_{2}^{*}$	Attractive in w where equation (17) holds Slow flow along center manifold towards p=0 if $R_{1}^{*}<R_{2}^{*}$
$p=1,\langle w,r\rangle /N=R_{1}^{*}$	Attractive in w where equation (17) holds Slow flow along center manifold towards p=1 if $R_{1}^{*}>R_{2}^{*}$
p=0,w=0	Saddle if $\tau (\alpha -1)<1/\|r{\|}_{1}$, otherwise stable
p=1,w=0	Saddle if $\tau (\alpha -1)<1/\|r{\|}_{1}$, otherwise stable
$p=\frac{R_{2}^{*}}{R_{1}^{*}+R_{2}^{*}},w=0$	Unstable if $\tau (\alpha -1)<1/\|r{\|}_{1}$, otherwise saddle
p=0,w=1	Additive: not an equilibrium Symmetric: unstable if $\tau (\alpha -1)<1/\|r{\|}_{1}$, otherwise saddle
$p=\frac{R_{2}^{*}-\|r{\|}_{1}/N}{R_{1}^{*}+R_{2}^{*}-2\|r{\|}_{1}/N},w=1$	Additive: not an equilibrium Symmetric: saddle if $\tau (\alpha -1)<1/\|r{\|}_{1}$, otherwise saddle
p=1,w=1	Additive: not an equilibrium Symmetric: unstable if $\tau (\alpha -1)<1/\|r{\|}_{1}$, otherwise saddle

## Footnotes

1 We keep the additive model despite its problems because it provides a simple point of comparison; when we implement the model, we clip the weights to keep them between 0 and 1, which in theory could occur through some sort of biological mechanism that we do not consider here. With the weights thus constrained, the model in fact performs reasonably well in many cases.

## References

[1] Markram H., Lübke J., Frotscher M., Sakmann B.: Regulation of Synaptic Efficacy by Coincidence of Postsynaptic APs and EPSPs. Science 275(5297), 213–215 (1997) 10.1126/science.275.5297.213 8985014

[2] Bi G. q., Poo M. m.: Synaptic Modifications in Cultured Hippocampal Neurons: Dependence on Spike Timing, Synaptic Strength, and Postsynaptic Cell Type. Journal of Neuroscience 18(24), 10464–10472 (1998) 10.1523/JNEUROSCI.18-24-10464.1998 9852584 PMC6793365

[3] Frémaux N., Gerstner W.: Neuromodulated Spike-Timing-Dependent Plasticity, and Theory of Three-Factor Learning Rules. Frontiers in Neural Circuits 9 (2016) 10.3389/fncir.2015.00085 PMC4717313 26834568

[4] Gerstner W., Lehmann M., Liakoni V., Corneil D., Brea J.: Eligibility Traces and Plasticity on Behavioral Time Scales: Experimental Support of NeoHebbian Three-Factor Learning Rules. Frontiers in Neural Circuits 12 (2018) 10.3389/fncir.2018.00053 PMC6079224 30108488

[5] Schultz W.: Predictive Reward Signal of Dopamine Neurons. Journal of Neurophysiology 80(1), 1–27 (1998) 10.1152/jn.1998.80.1.1 9658025

[6] Schultz W., Romo R.: Dopamine neurons of the monkey midbrain: contingencies of responses to stimuli eliciting immediate behavioral reactions. Journal of Neurophysiology 63(3), 607–624 (1990) 10.1152/jn.1990.63.3.607 2329364

[7] Surmeier D.J., Shen W., Day M., Gertler T., Chan S., Tian X., Plotkin J.L.: The role of dopamine in modulating the structure and function of striatal circuits. Progress in brain research 183, 149 (2010) 10.1016/S0079-6123(10)83008-0 20696319 PMC4431764

[8] Montague P., Dayan P., Sejnowski T.: A framework for mesencephalic dopamine systems based on predictive Hebbian learning. The Journal of Neuroscience 16(5), 1936–1947 (1996) 10.1523/JNEUROSCI.16-05-01936.1996 8774460 PMC6578666

[9] Schultz W., Dayan P., Montague P.R.: A Neural Substrate of Prediction and Reward. Science 275(5306), 1593–1599 (1997) 10.1126/science.275.5306.1593 9054347

[10] Lerner T.N., Holloway A.L., Seiler J.L.: Dopamine, Updated: Reward Prediction Error and Beyond. Current Opinion in Neurobiology 67, 123–130 (2021) 10.1016/j.conb.2020.10.012 33197709 PMC8116345

[11] Gurney K.N., Humphries M.D., Redgrave P.: A new framework for corticostriatal plasticity: behavioural theory meets in vitro data at the reinforcement-action interface. PLoS biology 13(1), 1002034 (2015) 10.1371/journal.pbio.1002034 PMC4285402 25562526

[12] Mikhael J.G., Bogacz R.: Learning reward uncertainty in the basal ganglia. PLoS computational biology 12(9), 1005062 (2016) 10.1371/journal.pcbi.1005062 PMC5010205 27589489

[13] Baladron J., Hamker F.H.: Habit learning in hierarchical cortex–basal ganglia loops. European Journal of Neuroscience 52(12), 4613–4638 (2020) 32237250 10.1111/ejn.14730

[14] Vich C., Dunovan K., Verstynen T., Rubin J.: Corticostriatal synaptic weight evolution in a two-alternative forced choice task: a computational study. Communications in Nonlinear Science and Numerical Simulation 82, 105048 (2020) 10.1016/j.cnsns.2019.105048

[15] Perez S., Cui Y., Vignoud G., Perrin E., Mendes A., Zheng Z., Touboul J., Venance L.: Striatum expresses region-specific plasticity consistent with distinct memory abilities. Cell Reports 38(11), 110521 (2022) 10.1016/j.celrep.2022.110521 35294877

[16] Smith R., Musleh W., Akopian G., Buckwalter G., Walsh J.P.: Regional differences in the expression of corticostriatal synaptic plasticity. Neuroscience 106(1), 95–101 (2001) 10.1016/S0306-4522(01)00260-3 11564420

[17] Wang Y.: Differential effect of aging on synaptic plasticity in the ventral and dorsal striatum. Neurobiology of Learning and Memory 89(1), 70–75 (2008) 10.1016/j.nlm.2007.08.015 17942327

[18] Gerfen C.R.: The neostriatal mosaic. i. compartmental organization of projections from the striatum to the substantia nigra in the rat. Journal of Comparative Neurology 236(4), 454–476 (1985) 2414339 10.1002/cne.902360404

[19] Lynd-Balta E., Haber S.: Primate striatonigral projections: a comparison of the sensorimotor-related striatum and the ventral striatum. Journal of Comparative Neurology 345(4), 562–578 (1994) 7962700 10.1002/cne.903450407

[20] Dunovan K., Vich C., Clapp M., Verstynen T., Rubin J.: Reward-driven changes in striatal pathway competition shape evidence evaluation in decision-making. PLoS computational biology 15(5), 1006998 (2019) 10.1371/journal.pcbi.1006998 PMC6534331 31060045

[21] Clapp M., Bahuguna J., Giossi C., Rubin J., Verstynen T.V., Vich C.: CBGTPy: An extensible cortico-basal ganglia-thalamic framework for modeling biological decision making. bioRxiv (2024) 10.1101/2023.09.05.556301 PMC11731724 39808625

[22] Abbott L.F., Blum K.I.: Functional Significance of Long-Term Potentiation for Sequence Learning and Prediction. Cerebral Cortex 6(3), 406–416 (1996) 10.1093/cercor/6.3.406 8670667

[23] Gerstner W., Kempter R., van Hemmen J.L., Wagner H.: A neuronal learning rule for sub-millisecond temporal coding. Nature 383(6595), 76–78 (1996) 10.1038/383076a0 8779718

[24] Gütig R., Aharonov R., Rotter S., Sompolinsky H.: Learning Input Correlations through Nonlinear Temporally Asymmetric Hebbian Plasticity. Journal of Neuroscience 23(9), 3697–3714 (2003) 10.1523/JNEUROSCI.23-09-03697.2003 12736341 PMC6742165

[25] Kistler W.M., Hemmen J.L.v.: Modeling Synaptic Plasticity in Conjunction with the Timing of Pre- and Postsynaptic Action Potentials. Neural Computation 12(2), 385–405 (2000) 10.1162/089976600300015844 10636948

[26] Rubin J., Lee D.D., Sompolinsky H.: Equilibrium Properties of Temporally Asymmetric Hebbian Plasticity. Physical Review Letters 86(2), 364–367 (2001) 10.1103/PhysRevLett.86.364 11177832

[27] Rossum M.C.W., Bi G.Q., Turrigiano G.G.: Stable Hebbian Learning from Spike Timing-Dependent Plasticity. Journal of Neuroscience 20(23), 8812–8821 (2000) 10.1523/JNEUROSCI.20-23-08812.2000 11102489 PMC6773092

[28] Richfield E.K., Penney J.B., Young A.B.: Anatomical and affinity state comparisons between dopamine d1 and d2 receptors in the rat central nervous system. Neuroscience 30(3), 767–777 (1989) 2528080 10.1016/0306-4522(89)90168-1

[29] Shen W., Flajolet M., Greengard P., Surmeier D.J.: Dichotomous Dopaminergic Control of Striatal Synaptic Plasticity. Science 321(5890), 848–851 (2008) 10.1126/science.1160575 18687967 PMC2833421

[30] Dreyer J.K., Herrik K.F., Berg R.W., Hounsgaard J.D.: Influence of phasic and tonic dopamine release on receptor activation. Journal of Neuroscience 30(42), 14273–14283 (2010) 20962248 10.1523/JNEUROSCI.1894-10.2010 PMC6634758

[31] Keeler J., Pretsell D., Robbins T.: Functional implications of dopamine D1 vs. D2 receptors: A ‘prepare and select’model of the striatal direct vs. indirect pathways. Neuroscience 282, 156–175 (2014) 25062777 10.1016/j.neuroscience.2014.07.021

[32] Shan Q., Ge M., Christie M.J., Balleine B.W.: The Acquisition of Goal-Directed Actions Generates Opposing Plasticity in Direct and Indirect Pathways in Dorsomedial Striatum. Journal of Neuroscience 34(28), 9196–9201 (2014) 10.1523/JNEUROSCI.0313-14.2014 25009253 PMC6608360

[33] Fisher S.D., Robertson P.B., Black M.J., Redgrave P., Sagar M.A., Abraham W.C., Reynolds J.N.J.: Reinforcement determines the timing dependence of corticostriatal synaptic plasticity in vivo. Nature Communications 8(1), 334 (2017) 10.1038/s41467-017-00394-x PMC5571189 28839128

[34] Shindou T., Shindou M., Watanabe S., Wickens J.: A silent eligibility trace enables dopamine-dependent synaptic plasticity for reinforcement learning in the mouse striatum. European Journal of Neuroscience 49(5), 726–736 (2019) 10.1111/ejn.13921 29603470 PMC6585681

[35] Houk J.C., Adams J.L., Barto A.G.: A Model of How the Basal Ganglia Generate and Use Neural Signals That Predict Reinforcement. In: Houk J.C., Davis J.L., Beiser D.G. (eds.) Models of Information Processing in the Basal Ganglia, pp. 249–270. The MIT Press, Cambridge, MA (1994). 10.7551/mitpress/4708.003.0020

[36] Sutton R.S., Barto A.G.: Reinforcement Learning, Second Edition: An Introduction. MIT Press, Cambridge, MA (2018)

[37] He K., Huertas M., Hong S.Z., Tie X., Hell J.W., Shouval H., Kirkwood A.: Distinct Eligibility Traces for LTP and LTD in Cortical Synapses. Neuron 88(3), 528–538 (2015) 10.1016/j.neuron.2015.09.037 26593091 PMC4660261

[38] Bogacz R.: Optimal decision-making theories: linking neurobiology with behaviour. Trends in Cognitive Sciences 11(3), 118–125 (2007) 10.1016/j.tics.2006.12.006 17276130

[39] Bogacz R., Gurney K.: The Basal Ganglia and Cortex Implement Optimal Decision Making Between Alternative Actions. Neural Computation 19(2), 442–477 (2007) 10.1162/neco.2007.19.2.442 17206871

[40] Vich C., Clapp M., Rubin J.E., Verstynen T.: Identifying control ensembles for information processing within the cortico-basal ganglia-thalamic circuit. PLoS Computational Biology 18(6), 1010255 (2022) 10.1371/journal.pcbi.1010255 PMC9258830 35737720

[41] Samejima K., Ueda Y., Doya K., Kimura M.: Representation of action-specific reward values in the striatum. Science 310(5752), 1337–1340 (2005) 16311337 10.1126/science.1115270

[42] Seo M., Lee E., Averbeck B.B.: Action selection and action value in frontal-striatal circuits. Neuron 74(5), 947–960 (2012) 22681697 10.1016/j.neuron.2012.03.037 PMC3372873

[43] Cisek P., Kalaska J.F.: Neural Correlates of Reaching Decisions in Dorsal Pre-motor Cortex: Specification of Multiple Direction Choices and Final Selection of Action. Neuron 45(5), 801–814 (2005) 10.1016/j.neuron.2005.01.027 15748854

[44] Rubin J.E., Vich C., Clapp M., Noneman K., Verstynen T.: The credit assignment problem in cortico-basal ganglia-thalamic networks: A review, a problem and a possible solution. European Journal of Neuroscience 53(7), 2234–2253 (2021) 10.1111/ejn.14745 32302439

[45] Bi G. q., Poo M. m.: Synaptic Modification by Correlated Activity: Hebb’s Postulate Revisited. Annual Review of Neuroscience 24(1), 139–166 (2001) 10.1146/annurev.neuro.24.1.139 11283308

[46] Riley B., Gould E., Lloyd J., Hallum L.E., Vlajkovic S., Todd K., Freestone P.S.: Dopamine transmission in the tail striatum: Regional variation and contribution of dopamine clearance mechanisms. Journal of Neurochemistry 168(3), 251–268 (2024) 10.1111/jnc.16052 38308566

[47] Yagishita S., Hayashi-Takagi A., Ellis-Davies G.C.R., Urakubo H., Ishii S., Kasai H.: A critical time window for dopamine actions on the structural plasticity of dendritic spines. Science 345(6204), 1616–1620 (2014) 10.1126/science.1255514 25258080 PMC4225776

[48] Virtanen P., Gommers R., Oliphant T.E., Haberland M., Reddy T., Cournapeau D., Burovski E., Peterson P., Weckesser W., Bright J., van der Walt S.J., Brett M., Wilson J., Millman K.J., Mayorov N., Nelson A.R.J., Jones E., Kern R., Larson E., Carey C.J., Polat İ., Feng Y., Moore E.W., Vander-Plas J., Laxalde D., Perktold J., Cimrman R., Henriksen I., Quintero E.A., Harris C.R., Archibald A.M., Ribeiro A.H., Pedregosa F., van Mulbregt P., SciPy 1.0 Contributors: SciPy 1.0: Fundamental Algorithms for Scientific Computing in Python. Nature Methods 17, 261–272 (2020) 10.1038/s41592-019-0686-2 32015543 PMC7056644

[49] Beron C.C., Neufeld S.Q., Linderman S.W., Sabatini B.L.: Mice exhibit stochastic and efficient action switching during probabilistic decision making. Proceedings of the National Academy of Sciences 119(15), 2113961119 (2022) 10.1073/pnas.2113961119 PMC9169659 35385355

[50] Bond K., Dunovan K., Porter A., Rubin J.E., Verstynen T.: Dynamic decision policy reconfiguration under outcome uncertainty. eLife 10, 65540 (2021) 10.7554/eLife.65540 PMC8806193 34951589

[51] Otani S., Daniel H., Roisin M.-P., Crepel F.: Dopaminergic modulation of long-term synaptic plasticity in rat prefrontal neurons. Cerebral Cortex 13(11), 1251–1256 (2003) 14576216 10.1093/cercor/bhg092

[52] Mendes A., Vignoud G., Perez S., Perrin E., Touboul J., Venance L.: Con-current thalamostriatal and corticostriatal spike-timing-dependent plasticity and heterosynaptic interactions shape striatal plasticity map. Cerebral Cortex 30(8), 4381–4401 (2020) 32147733 10.1093/cercor/bhaa024

[53] Kato S., Nishizawa K., Kobayashi K.: Thalamostriatal system controls the acquisition, performance, and flexibility of learning behavior. Frontiers in Systems Neuroscience 15, 729389 (2021) 34733142 10.3389/fnsys.2021.729389 PMC8558393

[54] Xie X., Seung H.S.: Learning in neural networks by reinforcement of irregular spiking. Physical Review E 69(4), 041909 (2004) 10.1103/PhysRevE.69.041909 15169045

[55] Izhikevich E.M.: Solving the Distal Reward Problem through Linkage of STDP and Dopamine Signaling. Cerebral Cortex 17(10), 2443–2452 (2007) 10.1093/cercor/bhl152 17220510

[56] Wärnberg E., Kumar A.: Feasibility of dopamine as a vector-valued feedback signal in the basal ganglia. Proceedings of the National Academy of Sciences 120(32), 2221994120 (2023) 10.1073/pnas.2221994120 PMC10410740 37527344

[57] O’Doherty J., Dayan P., Schultz J., Deichmann R., Friston K., Dolan R.J.: Dissociable Roles of Ventral and Dorsal Striatum in Instrumental Conditioning. Science 304(5669), 452–454 (2004) 10.1126/science.1094285 15087550

[58] Sosis B.: A Mathematical Analysis of Effective Learning and Decision-Making in Neuronal Circuits. PhD thesis, University of Pittsburgh (December 2024). https://d-scholarship.pitt.edu/47171/

[59] Sedrakyan H., Sedrakyan N.: Algebraic Inequalities. Problem Books in Mathematics. Springer, Cham (2018). 10.1007/978-3-319-77836-5

