# Supplementary material for: Distinct dopaminergic spike-timing-dependent plasticity rules are suited to different functional roles
Source: Res Sq. 2025 Sep 26:rs.3.rs-7456628. Preprint. [Version 1] doi: 10.21203/rs.3.rs-7456628/v1 (PMC12486093; doi:10.21203/rs.3.rs-7456628/v1)
Supplement: 1 [file NIHPPRS7456628V1-supplement-1.pdf]

Appendix A Averaged Model, Value Estimation Setting

A.1 Additive and Symmetric Models

Here we derive an averaged weight change model that adds up all contributions of pre-post and post-pre spike pairs and takes the average over realizations of the pre- and postsynaptic spike trains and over the possible timings of the dopamine signal, focusing on the additive and symmetric models in the value estimation setting. The presentation here largely follows that in [24], but with dopamine now included. (Much of the analysis also applies to the multiplicative model described in Section 2.1, but we do not focus on it here. For an analysis of the multiplicative model in a related setting, see [58].) We first give an expression for the total change in weight induced by a single triplet of a presynaptic spike at t_{pre}, a postsynaptic spike at t_{post}, and a dopamine signal D at t_{dop}. This can be found by integrating over the time since the largest of t_{pre}, t_{post}, and t_{dop}, because prior to t_{pre} or t_{post}, the eligibility trace is zero, and prior to t_{dop}, the dopamine trace is zero. The result for the additive and

symmetric models takes the form

$$\Delta w = \lambda D \int_{\max\{t_{\text{dop}}, t_{\text{pre}}, t_{\text{post}}\}}^{\infty} e^{-\frac{t-t_{\text{dop}}}{\tau_{\text{dop}}}} e^{-\frac{t-\max\{t_{\text{pre}}, t_{\text{post}}\}}{\tau_{\text{eli}}}} e^{-\frac{|t_{\text{post}}-t_{\text{pre}}|}{\tau}}$$
$$\times \left(\begin{cases} -f_-(w) & \text{if } t_{\text{post}} \leq t_{\text{pre}} \\ f_+(w) & \text{if } t_{\text{post}} > t_{\text{pre}} \end{cases} \right) dt$$
$$= \lambda D \frac{\tau_{\text{dop}} \tau_{\text{eli}}}{\tau_{\text{dop}} + \tau_{\text{eli}}} e^{-\frac{|t_{\text{post}}-t_{\text{pre}}|}{\tau}} \left(\begin{cases} -f_-(w) & \text{if } t_{\text{post}} \leq t_{\text{pre}} \\ f_+(w) & \text{if } t_{\text{post}} > t_{\text{pre}} \end{cases} \right)$$
$$\times \left(\begin{cases} e^{-\frac{|t_{\text{dop}}-\max\{t_{\text{pre}}, t_{\text{post}}\}|}{\tau_{\text{dop}}}} & \text{if } t_{\text{dop}} \leq \max\{t_{\text{pre}}, t_{\text{post}}\} \\ e^{-\frac{|t_{\text{dop}}-\max\{t_{\text{pre}}, t_{\text{post}}\}|}{\tau_{\text{eli}}}} & \text{if } t_{\text{dop}} > \max\{t_{\text{pre}}, t_{\text{post}}\} \end{cases} \right). \tag{A1}$$

We restate here the definition of the dopamine signal for the value estimation setting:

$$D = R^* - \overline{R} \tag{A2}$$

where

$$\overline{R} = \frac{1}{T_{\text{win}}} \int_{t_{\text{dop}}-T_{\text{win}}-T_{\text{del}}}^{t_{\text{dop}}-T_{\text{del}}} \rho^{\text{post}}(t)\, dt \tag{A3}$$

and R^* depends on the choice of action.

Following [24], we will define the cross-correlation functions $\Gamma_{i,\text{post}}(\Delta t) = \langle \rho_i^{\text{pre}}(t) \rho^{\text{post}}(t + \Delta t) \rangle_t$, where $\langle \cdot \rangle_t$ denotes averaging over time. These will arise in the averaging process. We also define the point process ρ^{dop} indicating when a dopamine signal is delivered, with rate $\langle \rho^{\text{dop}}(t) \rangle_t = r^{\text{dop}}$. (As noted previously, in simulations we assume for simplicity that dopamine is delivered periodically, but the precise form of the dopamine process does not matter as long as it has the given mean rate, it is independent of the spike trains, and dopamine signals are far enough apart that their interactions can be neglected.) Treating Δw as a function of t_{pre}, t_{post}, and t_{dop}, we can write the mean weight drift as follows:

$$\dot{w}_i = \left\langle \int_{-\infty}^{\infty} \int_{-\infty}^{\infty} \Delta w_i(t,\, t + \Delta t,\, t + \Delta t + \Delta s) \right.$$
$$\left. \rho_i^{\text{pre}}(t) \rho^{\text{post}}(t + \Delta t) \rho^{\text{dop}}(t + \Delta t + \Delta s)\, d\Delta s\, d\Delta t \right\rangle_t \tag{A4}$$

where $t = t_{\text{pre}}$, $\Delta t = t_{\text{post}} - t_{\text{pre}}$, and $\Delta s = t_{\text{dop}} - t_{\text{post}}$.

Note that ρ^{dop} is independent of the other terms. Also, if T_{del} is large enough, we can assume that $\overline{R}$ (and hence D) is independent of ρ^{post} (and hence also of ρ_i^{pre}, as $\overline{R}$ only depends on ρ_i^{pre} through ρ^{post}), because any postsynaptic spikes counted by the integral in equation (A3) must occur at least T_{del} before the dopamine signal, and consequently, either the negative exponential in $|t_{\text{dop}} - \max\{t_{\text{pre}}, t_{\text{post}}\}|$ or the one in

$|t_{\text{post}} - t_{\text{pre}}|$ in equation (A1) will be very small. Thus, assuming D is independent of the other terms provides a very good approximation if T_{del} is large enough. Another simplifying assumption we will make is that the weights change only a small amount on each dopamine release, so that we can treat w_i as constant in these expressions. Under these assumptions, we can substitute equations (A1) to (A3) into equation (A4) and split it into the $t_{\text{post}} \leq t_{\text{pre}}$ and $t_{\text{post}} > t_{\text{pre}}$ cases as follows:

$$\begin{aligned}
\dot{w}_i = -\lambda f_-(w_i) \int_{-\infty}^{0}\int_{-\infty}^{\infty} & \left(\langle R^* \rangle_t - \frac{1}{T_{\text{win}}} \int_0^{T_{\text{win}}} \left\langle \rho^{\text{post}}(u + t + \Delta t + \Delta s - T_{\text{del}} - T_{\text{win}}) \right\rangle_t du \right) \\
& \times \frac{\tau_{\text{dop}}\tau_{\text{eli}}}{\tau_{\text{dop}} + \tau_{\text{eli}}} e^{-\frac{|\Delta t|}{\tau}} \left(\begin{cases} e^{-\frac{|\Delta s + \Delta t|}{\tau_{\text{dop}}}} & \text{if } \Delta s + \Delta t \leq 0 \\ e^{-\frac{|\Delta s + \Delta t|}{\tau_{\text{eli}}}} & \text{if } \Delta s + \Delta t > 0 \end{cases} \right) \\
& \times \left\langle \rho_i^{\text{pre}}(t) \rho^{\text{post}}(t + \Delta t) \right\rangle_t \left\langle \rho^{\text{dop}}(t + \Delta t + \Delta s) \right\rangle_t \, d\Delta s \, d\Delta t \\
+ \lambda f_+(w_i) \int_{0}^{\infty}\int_{-\infty}^{\infty} & \left(\langle R^* \rangle_t - \frac{1}{T_{\text{win}}} \int_0^{T_{\text{win}}} \left\langle \rho^{\text{post}}(u + t + \Delta t + \Delta s - T_{\text{del}} - T_{\text{win}}) \right\rangle_t du \right) \\
& \times \frac{\tau_{\text{dop}}\tau_{\text{eli}}}{\tau_{\text{dop}} + \tau_{\text{eli}}} e^{-\frac{|\Delta t|}{\tau}} \left(\begin{cases} e^{-\frac{|\Delta s|}{\tau_{\text{dop}}}} & \text{if } \Delta s \leq 0 \\ e^{-\frac{|\Delta s|}{\tau_{\text{eli}}}} & \text{if } \Delta s > 0 \end{cases} \right) \\
& \times \left\langle \rho_i^{\text{pre}}(t) \rho^{\text{post}}(t + \Delta t) \right\rangle_t \left\langle \rho^{\text{dop}}(t + \Delta t + \Delta s) \right\rangle_t \, d\Delta s \, d\Delta t.
\end{aligned} \tag{A5}$$

We can write $\langle R^* \rangle_t$ as

$$\langle R^* \rangle_t = pR_1^* + (1 - p)R_2^* \tag{A6}$$

where p denotes the probability of selecting action A_1. We will derive dynamics for p below; for now we simply assume that, like w, it changes only a small amount on each dopamine release and so can be treated as a constant here.

Recall that the postsynaptic firing rate is given by

$$R(t) = \frac{1}{N} \sum_{i=1}^{N} w_i(t) \rho_i^{\text{pre}}(t - \epsilon).$$

It follows that for any x,

$$\begin{aligned}
\left\langle \rho^{\text{post}}(t + x) \right\rangle_t &= \frac{1}{N} \sum_{i=1}^{N} w_i \langle \rho_i^{\text{pre}}(t + x - \epsilon) \rangle_t \\
&= \frac{1}{N} \sum_{i=1}^{N} w_i r_i.
\end{aligned} \tag{A7}$$

In particular equation (A7) applies to $\langle \rho^{\text{post}}(u+t+\Delta t+\Delta s-T_{\text{del}}-T_{\text{win}})\rangle_t$ in equation (A5). Additionally, $\left\langle \rho^{\text{dop}}(t+\Delta t+\Delta s)\right\rangle_t = r^{\text{dop}}$ is a constant. We can therefore make the change of variables $\Delta s \leftarrow \Delta s + \Delta t$ to combine the positive and negative integrals, arriving at the formula:

$$\begin{aligned}
\dot{w}_i &= \left(pR_1^* + (1-p)R_2^* - \frac{1}{N}\sum_{i=1}^{N} w_i r_i \right) r^{\text{dop}} \frac{\tau_{\text{dop}}\tau_{\text{eli}}}{\tau_{\text{dop}}+\tau_{\text{eli}}} \int_{-\infty}^{\infty} \left(\begin{cases} e^{-\frac{|\Delta s|}{\tau_{\text{dop}}}} & \text{if } \Delta s \le 0 \\ e^{-\frac{|\Delta s|}{\tau_{\text{eli}}}} & \text{if } \Delta s > 0 \end{cases} \right) d\Delta s \\
&\quad \times \int_{-\infty}^{\infty} e^{-\frac{|\Delta t|}{\tau}} \left(\begin{cases} -\lambda f_-(w_i) & \text{if } \Delta t \le 0 \\ \lambda f_+(w_i) & \text{if } \Delta t > 0 \end{cases} \right) \Gamma_{i,\text{post}}(\Delta t)\, d\Delta t \\
&= \left(pR_1^* + (1-p)R_2^* - \frac{1}{N}\sum_{i=1}^{N} w_i r_i \right) r^{\text{dop}} \tau_{\text{dop}}\tau_{\text{eli}} \\
&\quad \times \int_{-\infty}^{\infty} e^{-\frac{|\Delta t|}{\tau}} \left(\begin{cases} -\lambda f_-(w_i) & \text{if } \Delta t \le 0 \\ \lambda f_+(w_i) & \text{if } \Delta t > 0 \end{cases} \right) \Gamma_{i,\text{post}}(\Delta t)\, d\Delta t. \qquad \text{(A8)}
\end{aligned}$$

Note that the remaining integral is exactly the one found in [24]. Using equation (A7) and following [24], we decompose $\Gamma_{i,\text{post}}$ as

$$\Gamma_{i,\text{post}}(\Delta t) = \frac{1}{N}\sum_{j=1}^{N} w_j \langle \rho_i^{\text{pre}}(t)\rho_j^{\text{pre}}(t+\Delta t-\epsilon)\rangle_t$$

and define the normalized cross-correlation function

$$\Gamma_{ij}^0(t') = \frac{\langle \rho_i^{\text{pre}}(t)\rho_j^{\text{pre}}(t+t')\rangle_t}{r_i r_j} - 1.$$

(Note that [24] assumes all presynaptic firing rates are identical, and so uses r^2 in the denominator instead.) We also define the effective cross-correlation matrices $C^{\pm}$ with elements

$$C_{ij}^+ = \int_0^{\infty} \frac{1}{\tau} e^{-\frac{|\Delta t|}{\tau}} \Gamma_{ij}^0(\Delta t-\epsilon)\, d\Delta t$$

and similarly for C_{ij}^- (which integrates from $-\infty$ to 0). Then we can rewrite the integrals in terms of $C_{ij}^{\pm}$:

$$\begin{aligned}
\lambda f_+(w_i) \int_0^{\infty} e^{-\frac{|\Delta t|}{\tau}} \Gamma_{i,\text{post}}(\Delta t)\, d\Delta t &= \lambda f_+(w_i) \frac{1}{N}\sum_{j=1}^{N} w_j \tau r_i r_j \\
&\quad \times \left(1 + \int_0^{\infty} \frac{1}{\tau} e^{-\frac{|\Delta t|}{\tau}} \Gamma_{ij}^0(\Delta t-\epsilon)\, d\Delta t \right)
\end{aligned}$$

$$= \lambda f_+(w_i) \frac{1}{N} \sum_{j=1}^{N} w_j \tau r_i r_j \left(1 + C_{ij}^+\right)$$

and similarly for the negative terms. Like in [24], we assume $\Gamma_{ij}^0(t') = \frac{1}{\sqrt{r_i r_j}} c_{ij} \delta(t')$ for some constants $c_{ij} \geq 0$ (again extending their formula to non-identical presynaptic firing rates). Since the argument of $\Gamma_{ij}^0(\Delta t - \epsilon)$ is never zero when $\Delta t < 0$, it follows that $C_{ij}^- = 0$ and $C_{ij}^+ = \frac{1}{\tau\sqrt{r_i r_j}} c_{ij} e^{-\epsilon/\tau} \approx \frac{1}{\tau\sqrt{r_i r_j}} c_{ij}$. (We assume, as in [24], that ϵ is small enough that $e^{-\epsilon/\tau} \approx 1$.) For Possion spike trains, the constants c_{ij} equal 1 if the spike trains are identical (because the autocorrelation is $\langle \rho(t)\rho(t+t')\rangle_t = r^2 + r\delta(t')$ for a Poisson spike train ρ with rate r) and are otherwise less than 1. We will assume that the presynaptic spike trains are uncorrelated, so $c_{ij} = 0$ for $i \neq j$. Therefore the formulas simplify as follows:

$$\lambda f_+(w_i) \frac{1}{N} \sum_{j=1}^{N} w_j \tau r_i r_j \left(1 + C_{ij}^+\right) = \lambda f_+(w_i) \frac{1}{N} \left(w_i r_i + \sum_{j=1}^{N} w_j \tau r_i r_j \right)$$

and

$$-\lambda f_-(w_i) \frac{1}{N} \sum_{j=1}^{N} w_j \tau r_i r_j \left(1 + C_{ij}^-\right) = -\lambda f_-(w_i) \frac{1}{N} \sum_{j=1}^{N} w_j \tau r_i r_j.$$

Substituting these results back into equation (A8), we obtain the formula for $\dot{w}_i$:

$$\dot{w}_i = \left(pR_1^* + (1-p)R_2^* - \frac{1}{N}\sum_{i=1}^{N} w_i r_i \right) r^{\text{dop}} \tau_{\text{dop}} \tau_{\text{eli}} \frac{\lambda}{N} \left(\tau \Delta f(w_i) r_i \left(\sum_{j=1}^{N} w_j r_j \right) + f_+(w_i) w_i r_i \right)$$

where $\Delta f = (f_+ - f_-)$. In vector notation, this formula can be written as:

$$\dot{w} = \left(pR_1^* + (1-p)R_2^* - \frac{1}{N}\langle w, r\rangle \right) r^{\text{dop}} \tau_{\text{dop}} \tau_{\text{eli}} \frac{\lambda}{N} \left(\tau \langle w, r\rangle \Delta f(w) \odot r + f_+(w) \odot w \odot r \right) \tag{A9}$$

where $\odot$ is the entrywise or Hadamard product and we treat $f_\pm(w)$ as applying entrywise.

We now derive dynamics for p, the probability of picking action A_1. Recall that $\overline{R}_1$ and $\overline{R}_2$ obey

$$\frac{d\overline{R}_j}{dt} = (-1)^{j-1} \frac{\overline{\lambda}}{2} D(t) \overline{A}(t) \tag{A10}$$

where $\overline{A}(t)$ indicates the previous action selected, 1 for A_1 and -1 for A_2. The change in $\overline{R}_j$ for a single dopamine release is then given by:

$$\Delta\overline{R}_j = (-1)^{j-1}\frac{\overline{\lambda}}{2}\overline{A}(t_{\text{dop}})\int_{t_{\text{dop}}}^{\infty} D(t)\,dt.$$

Taking an average only at the times dopamine is released, we see that

$$\langle\overline{A}(t_{\text{dop}})D(t_{\text{dop}})\rangle_{t_{\text{dop}}} = p\left(R_1^* - \frac{\langle w,r\rangle}{N}\right) - (1-p)\left(R_2^* - \frac{\langle w,r\rangle}{N}\right)$$

using equation (A6) and equation (A7) as above, assuming that p and w change only a small amount on each dopamine release. Incorporating the exponential decay of the dopamine signal over time, we get the average drift equation

$$\begin{aligned}\dot{\overline{R}}_j &= (-1)^{j-1}\frac{\overline{\lambda}}{2}r^{\text{dop}}\left(\int_0^{\infty} e^{-s/\tau_{\text{dop}}}\,ds\right)\left(p\left(R_1^* - \frac{\langle w,r\rangle}{N}\right) - (1-p)\left(R_2^* - \frac{\langle w,r\rangle}{N}\right)\right)\\ &= (-1)^{j-1}\frac{\overline{\lambda}}{2}r^{\text{dop}}\tau_{\text{dop}}\left(p\left(R_1^* - \frac{\langle w,r\rangle}{N}\right) - (1-p)\left(R_2^* - \frac{\langle w,r\rangle}{N}\right)\right).\end{aligned}$$

We can use this to derive an equation for p. From equation (7), we know that

$$p = \frac{1}{1+\exp\left(-\beta\left(\overline{R}_1 - \overline{R}_2\right)\right)},$$

the standard logistic function applied to the difference $\beta\left(\overline{R}_1 - \overline{R}_2\right)$. It follows that

$$\begin{aligned}\dot{p} &= p(1-p)\frac{d}{dt}\beta\left(\overline{R}_1 - \overline{R}_2\right)\\ &= \overline{\lambda}\beta r^{\text{dop}}\tau_{\text{dop}}p(1-p)\left(p\left(R_1^* - \frac{\langle w,r\rangle}{N}\right) - (1-p)\left(R_2^* - \frac{\langle w,r\rangle}{N}\right)\right).\end{aligned} \tag{A11}$$

Thus we end up with an $N+1$-dimensional system of differential equations for the N components of w along with p, given by equations (A9) and (16).

A.2 Corticostriatal Model

The analogous expression to equation (A1) for the corticostriatal model is:

$$\begin{aligned}\Delta w = \lambda\frac{\tau_{\text{dop}}\tau_{\text{eli}}}{\tau_{\text{dop}}+\tau_{\text{eli}}}e^{-\frac{|t_{\text{post}}-t_{\text{pre}}|}{\tau}}&\left(\begin{cases}-\alpha|D|w & \text{if } D(t_{\text{post}}-t_{\text{pre}}) \le 0\\ |D|(1-w) & \text{if } D(t_{\text{post}}-t_{\text{pre}}) > 0\end{cases}\right)\\ \times&\left(\begin{cases}e^{-\frac{|t_{\text{dop}}-\max\{t_{\text{pre}},t_{\text{post}}\}|}{\tau_{\text{dop}}}} & \text{if } t_{\text{dop}} \le \max\{t_{\text{pre}},t_{\text{post}}\}\\ e^{-\frac{|t_{\text{dop}}-\max\{t_{\text{pre}},t_{\text{post}}\}|}{\tau_{\text{eli}}}} & \text{if } t_{\text{dop}} > \max\{t_{\text{pre}},t_{\text{post}}\}\end{cases}\right).\end{aligned}$$

To derive an averaged form of the corticostriatal model, we need to decompose the expected dopamine signal into $\mathbb{E}[D] = D_+ + D_-$, where

$$\begin{aligned} D_+ &= \mathbb{E}[D \mid D \geq 0] P(D \geq 0) \\ D_- &= \mathbb{E}[D \mid D < 0] P(D < 0). \end{aligned}$$

These can be computed by counting the number of postsynaptic spikes to fall inside the window in equation (A3), using the cumulative distribution function of the Poisson distribution and averaging over the two actions; on the $D \geq 0$ side,

$$\begin{aligned} D_+ = p &\sum_{n=0}^{\lfloor R_1^* T_{\text{win}} \rfloor} \left(R_1^* - \frac{n}{T_{\text{win}}} \right) \frac{\left(r^{\text{post}} T_{\text{win}}\right)^n e^{-r^{\text{post}} T_{\text{win}}}}{n!} \\ &+ (1-p) \sum_{n=0}^{\lfloor R_2^* T_{\text{win}} \rfloor} \left(R_2^* - \frac{n}{T_{\text{win}}} \right) \frac{\left(r^{\text{post}} T_{\text{win}}\right)^n e^{-r^{\text{post}} T_{\text{win}}}}{n!} \\ &= p \left(R_1^* \sum_{n=0}^{\lfloor R_1^* T_{\text{win}} \rfloor} \frac{\left(r^{\text{post}} T_{\text{win}}\right)^n e^{-r^{\text{post}} T_{\text{win}}}}{n!} - r^{\text{post}} \sum_{n=1}^{\lfloor R_1^* T_{\text{win}} \rfloor} \frac{\left(r^{\text{post}} T_{\text{win}}\right)^{n-1} e^{-r^{\text{post}} T_{\text{win}}}}{(n-1)!} \right) \\ &+ (1-p) \left(R_2^* \sum_{n=0}^{\lfloor R_2^* T_{\text{win}} \rfloor} \frac{\left(r^{\text{post}} T_{\text{win}}\right)^n e^{-r^{\text{post}} T_{\text{win}}}}{n!} - r^{\text{post}} \sum_{n=1}^{\lfloor R_2^* T_{\text{win}} \rfloor} \frac{\left(r^{\text{post}} T_{\text{win}}\right)^{n-1} e^{-r^{\text{post}} T_{\text{win}}}}{(n-1)!} \right) \\ &= p \left(R_1^* \frac{\Gamma\left(\lfloor R_1^* T_{\text{win}} \rfloor + 1,\, r^{\text{post}} T_{\text{win}}\right)}{\Gamma\left(\lfloor R_1^* T_{\text{win}} \rfloor + 1\right)} - r^{\text{post}} \frac{\Gamma\left(\lfloor R_1^* T_{\text{win}} \rfloor,\, r^{\text{post}} T_{\text{win}}\right)}{\Gamma\left(\lfloor R_1^* T_{\text{win}} \rfloor\right)} \right) \\ &+ (1-p) \left(R_2^* \frac{\Gamma\left(\lfloor R_2^* T_{\text{win}} \rfloor + 1,\, r^{\text{post}} T_{\text{win}}\right)}{\Gamma\left(\lfloor R_2^* T_{\text{win}} \rfloor + 1\right)} - r^{\text{post}} \frac{\Gamma\left(\lfloor R_2^* T_{\text{win}} \rfloor,\, r^{\text{post}} T_{\text{win}}\right)}{\Gamma\left(\lfloor R_2^* T_{\text{win}} \rfloor\right)} \right) \end{aligned} \tag{A12}$$

where $r^{\text{post}} = \langle w, r \rangle / N$ is the postsynaptic firing rate. Since $\mathbb{E}[D] = pR_1^* + (1-p)R_2^* - r^{\text{post}}$, it follows that $D_- = pR_1^* + (1-p)R_2^* - r^{\text{post}} - D_+$. Then an analogous derivation to that in Appendix A.1, treating the $D \geq 0$ and $D < 0$ cases separately, gives the following average weight drift formula

$$\begin{aligned} \dot{w} = r^{\text{dop}} \tau_{\text{dop}} \tau_{\text{eli}} \frac{\lambda}{N} \Big(& D_+ \left(\tau \langle w, r \rangle \left(1 - (1+\alpha) w\right) \odot r + (1-w) \odot w \odot r \right) \\ & - D_- \left(\tau \langle w, r \rangle \left(1 - (1+\alpha) w\right) \odot r - \alpha w \odot w \odot r \right) \Big), \end{aligned} \tag{A13}$$

with $\dot{p}$ obeying equation (A11).

Appendix B Analysis of Equilibria, Value Estimation Setting

B.1 Equilibria with $p = 0$ or $p = 1$

It is clear from equations (A9) and (16) that the following sets of points are equilibria for the additive and symmetric models:

$$
\begin{aligned}
p &= 0,\ \frac{\langle w, r\rangle}{N} = R_2^* \\
p &= 1,\ \frac{\langle w, r\rangle}{N} = R_1^* \\
p &= \frac{R_2^*}{R_1^* + R_2^*},\ 0,\ \text{or } 1,\ w = \mathbf{0}.
\end{aligned}
$$

We now analyze the first two sets of points, which define $N-1$-dimensional hyperplanes of weights. Assuming that $R_1^* > R_2^*$, so that action A_1 is more rewarding than A_2, we would like to find conditions such that p converges to 1 and w converges to a point on the hyperplane $\langle w, r\rangle/N = R_1^*$, indicating that the system is correctly predicting the rewards it is receiving. (In the following analysis we generally assume for simplicity that each r_i is nonzero, since the w_i coordinate becomes trivial if $r_i = 0$.)

Theorem 2 *Pick w and p so that either $p = 1$, $\langle w, r\rangle/N = R_1^*$ or $p = 0$, $\langle w, r\rangle/N = R_2^*$. For the additive and symmetric models given by equations (A9) and (16), the Jacobian at this equilibrium has one nonzero eigenvalue, which has an eigenvector that is zero in the p coordinate. This eigenvalue is negative if and only if*

$$
0 < \sum_{i=1}^{N} r_i^2 \left(\tau N R^* \Delta f(w_i) + f_+(w_i) w_i\right), \tag{17}
$$

with the choice of $R^* = R_1^*$ or $R^* = R_2^*$ corresponding to the equilibrium under examination.

Proof The Jacobian at these points can be computed as follows:

$$
\frac{\partial \dot{w}_i}{\partial w_j} = -\frac{r_j}{N} r^{\text{dop}} \tau_{\text{dop}} \tau_{\text{eli}} \frac{\lambda}{N} \left(\tau \langle w, r\rangle \Delta f(w_i) r_i + f_+(w_i) w_i r_i\right), \tag{B14}
$$

$$
\frac{\partial \dot{w}_i}{\partial p} = (R_1^* - R_2^*) r^{\text{dop}} \tau_{\text{dop}} \tau_{\text{eli}} \frac{\lambda}{N} \left(\tau \langle w, r\rangle \Delta f(w_i) r_i + f_+(w_i) w_i r_i\right), \tag{B15}
$$

$$
\begin{aligned}
\frac{\partial \dot{p}}{\partial w_j} &= \overline{\lambda} \beta r^{\text{dop}} \tau_{\text{dop}} p(1-p)(1-2p) \frac{r_j}{N} \\
&= 0, \\
\frac{\partial \dot{p}}{\partial p} &= \overline{\lambda} \beta r^{\text{dop}} \tau_{\text{dop}} \left(p(2-3p) \left(R_1^* - \frac{\langle w, r\rangle}{N} \right) + (1-p)(3p-1) \left(R_2^* - \frac{\langle w, r\rangle}{N} \right) \right) \\
&= 0.
\end{aligned}
$$

We will write the Jacobian matrix as

$$
J = \begin{pmatrix} J_w & \partial \dot{w}/\partial p \\ \mathbf{0}^T & 0 \end{pmatrix}
$$

where

$$J_w = -r^{\text{dop}}\tau_{\text{dop}}\tau_{\text{eli}}\frac{\lambda}{N^2}\left(\tau\langle w, r\rangle\Delta f(w)\odot r + f_+(w)\odot w\odot r\right)r^T.$$

The submatrix J_w has eigenvalue 0 with multiplicity $N-1$ corresponding to the subspace orthogonal to r, that is, parallel to the planes $\langle w, r\rangle/N = R_1^*, R_2^*$. Its remaining eigenvalue is given by

$$\Lambda = -r^{\text{dop}}\tau_{\text{dop}}\tau_{\text{eli}}\frac{\lambda}{N^2}\left\langle\tau\langle w, r\rangle\Delta f(w)\odot r + f_+(w)\odot w\odot r,\, r\right\rangle \tag{B16}$$

with associated eigenvector

$$v_1 = \tau\langle w, r\rangle\Delta f(w)\odot r + f_+(w)\odot w\odot r. \tag{B17}$$

In general, the condition $\Lambda < 0$ (indicating stability) will define a subset of the planes, the shape of which depends on the precise form of f_+ and f_-:

$$\Lambda < 0 \quad\Longleftrightarrow\quad 0 < \sum_{i=1}^{N} r_i^2\left(\tau N R^*\Delta f(w_i) + f_+(w_i)w_i\right)$$

where we have used the fact that $\langle w, r\rangle/N = R^*$ on the plane (with the choice of $R^* = R_1^*$ or $R^* = R_2^*$ depending on which equilibrium we are examining).

The full matrix J retains this eigenvalue (the associated eigenvector simply has 0 in the p dimension). The eigenvalue 0 has multiplicity N for J. Like J_w, it has $N-1$ eigenvectors orthogonal to r (with 0 in the p dimension) and additionally has the eigenvector

$$\left(r,\ \frac{\langle r, r\rangle}{(R_1^* - R_2^*)N}\right) \in \mathbb{R}^{N+1},$$

which is parallel to line segments stretching from the $p = 0$ hyperplane to the closest point on the $p = 1$ hyperplane. □

To summarize, at $p = 0$ and $p = 1$ there exist hyperplanes of equilibria defined by $\langle w, r\rangle/N = R_2^*$ and $\langle w, r\rangle/N = R_1^*$, respectively. The Jacobian on these planes has one nonzero eigenvalue, Λ, which determines the direction of flow off the plane in the w direction (with zero component in the p direction). For the additive model, $f_+ = 1$ and $f_- = \alpha$, so the stability condition found above can be rewritten as follows:

$$0 < \sum_{i=1}^{N} r_i^2\left(\tau N R^*(1-\alpha) + w_i\right) \quad\Longleftrightarrow\quad \|r\|_2^2\tau N R^*(\alpha - 1) < \langle r^2, w\rangle \tag{B18}$$

where r^2 is the vector consisting of elements r_i^2. This condition holds on a half-space with a boundary perpendicular to r^2 which will generally divide the hyperplanes of equilibria into two domains, one stable and one unstable. Note that if $\alpha = 1$ this condition is always true for $w_i \in (0, 1]$, so the entire plane is stable in this direction; however, in general we may have $\alpha > 1$, in which case this global stability will not hold.

For the symmetric model, $f_+(w) = w(1-w)$ and $f_- = \alpha w(1-w)$. The stability condition is therefore a cubic polynomial in w:

$$0 < \sum_{i=1}^{N} r_i^2 w_i(1-w_i)\left(\tau N R^*(1-\alpha) + w_i\right).$$

Like with the additive model, this condition is always satisfied when $\alpha = 1$ (unless $w_i = 0$ or $w_i = 1$ for all i). In general, though, the region where this condition is satisfied will be more complex.

At each of these equilibria there also exists a center eigenvector with a nonzero component in the p coordinate, which we call the nontrivial center manifold. We now examine the behavior of the system along the nontrivial center manifolds:

Theorem 3 *Points of the form $p = 0$, $\langle w, r\rangle/N = R_2^*$ or $p = 1$, $\langle w, r\rangle/N = R_1^*$ are equilibria of equations (A9) and (16) with a nontrivial center manifold; the flow along this manifold is towards $p = 1$ and away from $p = 0$ if $R_1^* > R_2^*$, or away from $p = 1$ and towards $p = 0$ if $R_1^* < R_2^*$.*

Proof We focus for now on the $p = 0$ case. Let w_0 be a point that satisfies $\langle w, r\rangle/N = R_2^*$, so that $(w_0, 0)$ is an equilibrium of equations (A9) and (16), and let $\hat{w} = w - w_0$. Let $v_1 \in \mathbb{R}^N$ be the eigenvector of J_w at the point $(w_0, 0)$ defined in equation (B17) (associated with the single nonzero eigenvalue), let $v_2, \ldots, v_N$ be vectors orthogonal to r (so that they span the zero eigenspace of J_w), and assume without loss of generality that $\langle v_i, v_j\rangle = \delta_{ij}$ for $i, j = 2, \ldots, N$ so that the vectors are orthonormal. Let $V_1 \in \mathbb{R}^{N\times N}$ be the matrix with columns $v_1, v_2, \ldots, v_N$. We can then use the following coordinate transformation to align the axes of the system with the eigenvectors of J:

$$\begin{pmatrix} \hat{w} \\ p \end{pmatrix} = \begin{pmatrix} V_1 & \frac{r}{\langle r,r\rangle} \\ \mathbf{0}^T & \frac{1}{N(R_1^* - R_2^*)} \end{pmatrix} \begin{pmatrix} x \\ y \end{pmatrix} \tag{B19}$$

where $x \in \mathbb{R}^N$ and y is a scalar multiple of p. Let $V \in \mathbb{R}^{(N+1)\times(N+1)}$ be the entire coordinate transform matrix.

The only nonzero eigenvalue is associated with the coordinate x_1, so the center manifold is of the form $x_1 = h(x_{2\cdots N}, y)$ (where $x_{2\cdots N} = (x_2, x_3, \ldots, x_N) \in \mathbb{R}^{N-1}$) such that $h(\mathbf{0}) = 0$ and $\nabla h(\mathbf{0}) = \mathbf{0}$. It follows that on the manifold,

$$\dot{x}_1 = \langle \nabla h, (\dot{x}_{2\cdots N}, \dot{y})\rangle . \tag{B20}$$

First note that using equation (B19),

$$\begin{aligned} \langle \hat{w}, r\rangle &= \langle V_1 x, r\rangle + y \\ &= \langle x, V_1^T r\rangle + y \\ &= \langle v_1, r\rangle x_1 + y, \end{aligned}$$

and by taking the time derivative we get

$$\dot{x}_1 = \frac{\langle \dot{w}, r\rangle - \dot{y}}{\langle v_1, r\rangle}$$

(since $\dot{\hat{w}} = \dot{w}$). To address the right-hand-side of equation (B20), we introduce the $N \times N$ matrix

$$V_2 = \begin{pmatrix} v_2 & v_3 & \cdots & v_N & \frac{r}{\langle r,r\rangle} \end{pmatrix} .$$

Since V_2 is orthonormal, we have

$$\begin{aligned} \langle \nabla h, (\dot{x}_{2\cdots N}, \dot{y})\rangle &= \langle V_2 \nabla h, V_2(\dot{x}_{2\cdots N}, \dot{y})\rangle \\ &= \langle V_2 \nabla h, \dot{w} - \dot{x}_1 v_1\rangle \end{aligned}$$

by taking the time derivative of the $\hat{w}$ coordinates of equation (B19). Combining these results, we obtain

$$\begin{aligned}\dot{x}_1 &= \langle V_2\nabla h,\, \dot{w} - \dot{x}_1 v_1\rangle \\ \iff \frac{\langle \dot{w},\, r\rangle - \dot{y}}{\langle v_1,\, r\rangle} &= \frac{\langle V_2\nabla h,\, \dot{w}\rangle}{1 + \langle V_2\nabla h,\, v_1\rangle} \\ \iff \langle V_2\nabla h,\, \dot{w}\rangle \langle v_1,\, r\rangle &= (1 + \langle V_2\nabla h,\, v_1\rangle)\,(\langle \dot{w},\, r\rangle - \dot{y})\,.\end{aligned}$$

Note that ∇h is first order, so the terms involving $\dot{w}$ or $\dot{y}$ multiplied by ∇h are strictly higher order than the terms involving only $\dot{w}$ or $\dot{y}$ and can be dropped, leaving:

$$\langle \dot{w},\, r\rangle = \dot{y} + \text{h.o.t.} \tag{B21}$$

(Here "h.o.t." stands for higher-order terms.)

We now collect the leading-order terms in this expression. To see the behavior of $\dot{w}$ near the equilibrium $(w, p) = (w_0, 0)$, where we recall that $w_0 = \langle w, r\rangle/N = R_2^*$, we substitute $w = w_0 + \hat{w} = w_0 + x_1 v_1 + V_2(x_{2\cdots N}, y)$ and $p = \frac{1}{N(R_1^* - R_2^*)} y$ into equation (A9) to compute

$$\begin{aligned}\dot{w} &= \left(pR_1^* + (1-p)R_2^* - \frac{1}{N}\langle \hat{w} + w_0, r\rangle\right) r^{\text{dop}} \tau_{\text{dop}} \tau_{\text{eli}} \frac{\lambda}{N} \\ &\quad \times \left(\tau\langle \hat{w} + w_0, r\rangle \Delta f(\hat{w} + w_0) \odot r + f_+(\hat{w} + w_0) \odot (\hat{w} + w_0) \odot r\right) \\ &= \left(p\left(R_1^* - R_2^*\right) - \frac{1}{N}\langle \hat{w}, r\rangle\right) r^{\text{dop}} \tau_{\text{dop}} \tau_{\text{eli}} \frac{\lambda}{N} \\ &\quad \times \left(\tau\left(\langle \hat{w}, r\rangle + NR_2^*\right) \Delta f(\hat{w} + w_0) \odot r + f_+(\hat{w} + w_0) \odot (\hat{w} + w_0) \odot r\right) \\ &= \left(\frac{y}{N} - \frac{1}{N}\langle v_1 h(x_{2\cdots N}, y) + V_2(x_{2\cdots N}, y), r\rangle\right) r^{\text{dop}} \tau_{\text{dop}} \tau_{\text{eli}} \frac{\lambda}{N} \\ &\quad \times \Big(\tau\left(\langle v_1 h(x_{2\cdots N}, y) + V_2(x_{2\cdots N}, y), r\rangle + NR_2^*\right) \Delta f\left(v_1 h(x_{2\cdots N}, y) + V_2(x_{2\cdots N}, y) + w_0\right) \odot r \\ &\qquad + f_+\left(v_1 h(x_{2\cdots N}, y) + V_2(x_{2\cdots N}, y) + w_0\right) \odot \left(v_1 h(x_{2\cdots N}, y) + V_2(x_{2\cdots N}, y) + w_0\right) \odot r\Big).\end{aligned}$$

We know that $\langle V_2(x_{2\cdots N}, y), r\rangle = y$, so this expression can be written

$$\begin{aligned}\dot{w} &= -h(x_{2\cdots N}, y)\langle v_1, r\rangle r^{\text{dop}} \tau_{\text{dop}} \tau_{\text{eli}} \frac{\lambda}{N^2} \\ &\quad \times \Big(\tau\left(h(x_{2\cdots N}, y)\langle v_1, r\rangle + y + NR_2^*\right) \Delta f\left(v_1 h(x_{2\cdots N}, y) + V_2(x_{2\cdots N}, y) + w_0\right) \odot r \\ &\qquad + f_+\left(v_1 h(x_{2\cdots N}, y) + V_2(x_{2\cdots N}, y) + w_0\right) \odot \left(v_1 h(x_{2\cdots N}, y) + V_2(x_{2\cdots N}, y) + w_0\right) \odot r\Big) \\ &= -h(x_{2\cdots N}, y)\langle v_1, r\rangle r^{\text{dop}} \tau_{\text{dop}} \tau_{\text{eli}} \frac{\lambda}{N^2}\left(\tau N R_2^* \Delta f(w_0) \odot r + f_+(w_0) \odot w_0 \odot r\right) + \text{h.o.t.}\end{aligned} \tag{B22}$$

Similarly,

$$\begin{aligned}\dot{p} &= \overline{\lambda}\beta r^{\text{dop}} \tau_{\text{dop}} p(1-p)\left(p\left(R_1^* - \frac{\langle \hat{w} + w_0, r\rangle}{N}\right) - (1-p)\left(R_2^* - \frac{\langle \hat{w} + w_0, r\rangle}{N}\right)\right) \\ &= \overline{\lambda}\beta r^{\text{dop}} \tau_{\text{dop}} p(1-p)\left(p\left(R_1^* - R_2^*\right) + (1-2p)\frac{\langle \hat{w}, r\rangle}{N}\right) \\ &= \overline{\lambda}\beta r^{\text{dop}} \tau_{\text{dop}} \frac{y}{N(R_1^* - R_2^*)}\left(1 - \frac{y}{N(R_1^* - R_2^*)}\right) \\ &\quad \times \left(\frac{y}{N} + \left(1 - \frac{2y}{N(R_1^* - R_2^*)}\right)\frac{h(x_{2\cdots N}, y)\langle v_1, r\rangle + y}{N}\right)\end{aligned}$$

$$= \overline{\lambda}\beta r^{\text{dop}}\tau_{\text{dop}} \frac{2y^2}{N^2(R_1^* - R_2^*)} + \text{h.o.t.} \tag{B23}$$

Moreover, equation (B23) implies that

$$\begin{aligned} \dot{y} &= N(R_1^* - R_2^*)\dot{p} \\ &= \overline{\lambda}\beta r^{\text{dop}}\tau_{\text{dop}} \frac{2y^2}{N} + \text{h.o.t.} \end{aligned} \tag{B24}$$

We can use equations (B22) and (B24) in conjunction with equation (B21) to derive an expression for h:

$$\begin{aligned} h(x_{2\cdots N}, y) &= -\frac{2N\overline{\lambda}\beta r^{\text{dop}}\tau_{\text{dop}}}{\langle v_1, r\rangle \lambda r^{\text{dop}}\tau_{\text{dop}}\tau_{\text{eli}} \left\langle \tau N R_2^* \Delta f(w_0) \odot r + f_+(w_0) \odot w_0 \odot r,\, r\right\rangle} y^2 + \text{h.o.t.} \\ &= \frac{2\overline{\lambda}\beta r^{\text{dop}}\tau_{\text{dop}}}{N\Lambda\langle v_1, r\rangle} y^2 + \text{h.o.t.} \end{aligned}$$

using the definitions of v_1 and Λ (equations (B16) and (B17)); we will abbreviate this as $h(x_{2\cdots N}, y) = cy^2 + \text{h.o.t.}$ Note that $c < 0$ because Λ has the opposite sign as $\langle v_1, r\rangle$.

Translating back into (w, p) coordinates, this means that we can parameterize the nontrivial center manifold near the equilibrium $(w_0, 0)$ as follows:

$$w = w_0 + \sum_{i=2}^{N} v_i x_i + \frac{r}{\langle r, r\rangle} y + \frac{2\overline{\lambda}\beta r^{\text{dop}}\tau_{\text{dop}}}{N\Lambda\langle v_1, r\rangle} v_1 y^2 + \text{h.o.t.} \tag{B25}$$

$$p = \frac{1}{N(R_1^* - R_2^*)} y. \tag{B26}$$

That is, the manifold consists of a linear term along the vector r, a quadratic term along the vector $-v_1$ (negative since Λ has the opposite sign as $\langle v_1, r\rangle$), and arbitrary translations orthogonal to r (from the $v_2, \ldots, v_N$ terms).

We can now determine the dynamics on the manifold near the equilibrium. First, the flow in the y direction is always positive by equation (B24). Since $y = N(R_1^* - R_2^*)p$ and $p \geq 0$, the sign of $\dot{p}$ is determined by the sign of $R_1^* - R_2^*$: if $R_1^* > R_2^*$, then $\dot{p} > 0$, implying that the flow is away from $p = 0$; if $R_1^* < R_2^*$, then $\dot{p} < 0$, implying that the flow is towards $p = 0$. Thus the flow along this manifold promotes selection of the action that leads to the larger reward.

Meanwhile, the flow in w is described by equation (B22), which can be rewritten as follows using the definitions of Λ and v_1:

$$\begin{aligned} \dot{w} &= h(x_{2\cdots N}, y)\Lambda v_1 + \text{h.o.t.} \\ &= \frac{2\overline{\lambda}\beta r^{\text{dop}}\tau_{\text{dop}}}{N\langle v_1, r\rangle} v_1 y^2 + \text{h.o.t.} \\ &= \frac{2\overline{\lambda}\beta r^{\text{dop}}\tau_{\text{dop}}}{\langle v_1, r\rangle} N(R_1^* - R_2^*)^2 v_1 p^2 + \text{h.o.t.} \end{aligned}$$

If $\langle v_1, r\rangle > 0$, then $\Lambda < 0$, so that trajectories near the equilibrium converge to it exponentially in the w axes, while on the manifold, there is a slow flow in the direction of v_1. If $\langle v_1, r\rangle < 0$, then trajectories diverge exponentially from the equilibrium, while on the manifold, the slow flow is in the direction of $-v_1$. In the $N = 1$ case shown in Figure 7, the sign of $\langle v_1, r\rangle$ simply equals the sign of the scalar v_1, so trajectories along the center manifold always increase in w when sufficiently close to the equilibrium.

The symmetry of the system makes analysis of the center manifold at the $p = 1$ equilibrium very similar to that of the $p = 0$ case. In fact, replacing p with $1 - p$ only has the effect of

swapping the roles of R_1^* and R_2^*. Concretely, we select w_0 such that $\langle w_0, r\rangle/N = R_1^*$ (so that the point $w = w_0$ lies on the hyperplane of equilibria associated with $p = 1$), let $\hat{p} = 1 - p$, and make the change of coordinates $y = N(R_2^* - R_1^*)\hat{p}$, with the order of R_1^* and R_2^* reversed. It can then be seen that the formula to parameterize the manifold near $(w, p) = (w_0, 1)$ is identical to equation (B25) for w (although w_0 is different, and Λ and v_1 also depend on the choice of w_0), while the expression for p is

$$p = 1 + \frac{1}{N(R_1^* - R_2^*)} y,$$

where in this case we will have $y < 0$ to parameterize values of p in $[0, 1]$. Similarly, the sign of $\dot{p}$ will be positive if $R_1^* > R_2^*$, but in this case that implies the flow will be *towards* $p = 1$, while $R_1^* < R_2^*$ implies the flow will be away from $p = 1$: in both cases the flow is towards picking the action that leads to the larger reward. □

B.2 Equilibria with $w = 0$

As mentioned previously, in addition to the equilibria at $p = 0$ and $p = 1$ there are also equilibria at $w = \mathbf{0}$ and $p = R_2^*/(R_1^* + R_2^*)$, 0, or 1. The Jacobian at each of these points depends on the choice of f_+ and f_-. We first analyze the additive model, where $f_+ = 1$ and $f_- = \alpha$.

Proposition 4 *For the additive model, if the condition*

$$\tau(\alpha - 1) < \frac{1}{\|r\|_1}. \tag{B27}$$

holds, then the $w = \mathbf{0}$, $p = 0$ and $w = \mathbf{0}$, $p = 1$ equilibria are saddles where the Jacobian has exactly one negative eigenvalue (corresponding to the eigenvector $(0, \ldots, 0, 1)$), with all other eigenvalues real and positive. They gain at least one zero or negative eigenvalue if the condition does not hold. The $w = \mathbf{0}$, $p = R_2^*/(R_1^* + R_2^*)$ equilibrium has a Jacobian with only positive real eigenvalues if equation (B27) holds, but gains at least one zero or negative eigenvalue if the condition fails.

Proof The Jacobian can be computed as follows:

$$\begin{aligned}
\frac{\partial \dot{w}_i}{\partial w_j} &= \left(pR_1^* + (1-p)R_2^*\right) r^{\text{dop}} \tau_{\text{dop}} \tau_{\text{eli}} \frac{\lambda}{N} \left(\tau(1-\alpha) r_i r_j + \delta_{ij} r_i\right), \\
\frac{\partial \dot{w}_i}{\partial p} &= 0, \\
\frac{\partial \dot{p}}{\partial w_j} &= \overline{\lambda} \beta r^{\text{dop}} \tau_{\text{dop}} p(1-p)(1-2p) \frac{r_j}{N}, \\
\frac{\partial \dot{p}}{\partial p} &= \overline{\lambda} \beta r^{\text{dop}} \tau_{\text{dop}} \left(p(2-3p)R_1^* + (1-p)(3p-1)R_2^*\right)
\end{aligned}$$

for $p = 0$, 1, $R_2^*/(R_1^* + R_2^*)$. We will write the Jacobian matrix as

$$J = \begin{pmatrix} J_w & \mathbf{0} \\ \partial \dot{p}/\partial w & \partial \dot{p}/\partial p \end{pmatrix}$$

where

$$J_w = \left(pR_1^* + (1-p)R_2^*\right) r^{\text{dop}} \tau_{\text{dop}} \tau_{\text{eli}} \frac{\lambda}{N} \left(\tau(1-\alpha) r r^T + \text{diag}(r)\right) \tag{B28}$$

is symmetric.

As J is block-triangular, one eigenvalue is given by $\partial \dot{p}/\partial p$, which varies depending on which equilibrium we are considering. The corresponding eigenvector is $(0,\ldots,0,1) \in \mathbb{R}^{N+1}$, pointing entirely along the p axis. The remainder of the eigenvalues are eigenvalues of J_w. We now show that J_w is positive definite if and only if the condition given in equation (B27) holds.

To see that this condition determines the definiteness of J_w, we examine $\langle x, J_w x\rangle$ for $x \in \mathbb{R}^N$:

$$\langle x, J_w x\rangle = \left(pR_1^* + (1-p)R_2^*\right) r^{\text{dop}} \tau_{\text{dop}} \tau_{\text{eli}} \frac{\lambda}{N} \left(\tau(1-\alpha)\left(\sum_{i=1}^N x_i r_i\right)^2 + \sum_{i=1}^N x_i^2 r_i\right).$$

We assume $r_i > 0$ for each i, which allows us to apply Sedrakyan's inequality[2] [59]:

$$\left(\sum_{i=1}^N x_i r_i\right)^2 \leq \left(\sum_{i=1}^N r_i\right)\left(\sum_{i=1}^N \frac{(x_i r_i)^2}{r_i}\right) = \|r\|_1 \left(\sum_{i=1}^N x_i^2 r_i\right).$$

Suppose that $\tau(\alpha - 1) < \|r\|_1^{-1}$. Then we have the bound:

$$\langle x, J_w x\rangle > \left(pR_1^* + (1-p)R_2^*\right) r^{\text{dop}} \tau_{\text{dop}} \tau_{\text{eli}} \frac{\lambda}{N} \left(-\frac{1}{\|r\|_1}\left(\sum_{i=1}^N x_i r_i\right)^2 + \sum_{i=1}^N x_i^2 r_i\right) \geq 0$$

by Sedrakyan's inequality, so J_w is positive definite. Conversely, if $\langle x, J_w x\rangle > 0$ for all x, then in particular it must hold for $x = \mathbf{1}$, so we get:

$$0 < \langle \mathbf{1}, J\mathbf{1}\rangle = \left(pR_1^* + (1-p)R_2^*\right) r^{\text{dop}} \tau_{\text{dop}} \tau_{\text{eli}} \frac{\lambda}{N} \left(\tau(1-\alpha)\|r\|_1^2 + \|r\|_1\right)$$

which can be rearranged to give $\tau(\alpha-1) < \|r\|_1^{-1}$. Thus we see that if equation (B27) holds, J_w only has positive eigenvalues, whereas if it does not hold, it must have at least one zero or negative eigenvalue.

The overall stability of the equilibria also depend on $\partial \dot{p}/\partial p$ as follows:

$$\begin{aligned}
\text{At } p = 0\text{:} \quad & \frac{\partial \dot{p}}{\partial p} = -\overline{\lambda}\beta r^{\text{dop}} \tau_{\text{dop}} R_2^* \\
\text{At } p = \frac{R_2^*}{R_1^* + R_2^*}\text{:} \quad & \frac{\partial \dot{p}}{\partial p} = \overline{\lambda}\beta r^{\text{dop}} \tau_{\text{dop}} \frac{R_1^* R_2^*}{R_1^* + R_2^*} \\
\text{At } p = 1\text{:} \quad & \frac{\partial \dot{p}}{\partial p} = -\overline{\lambda}\beta r^{\text{dop}} \tau_{\text{dop}} R_1^*
\end{aligned}$$

So the $w = \mathbf{0}, p = 0$ and $w = \mathbf{0}, p = 1$ equilibria are saddles with exactly one negative eigenvalue (corresponding to the eigenvector $(0,\ldots,0,1)$) if equation (B27) holds, and they gain at least one zero or negative eigenvalue if the condition does not hold. The $w = \mathbf{0}, p = R_2^*/(R_1^* + R_2^*)$ equilibrium is unstable (i.e. only positive eigenvalues) if equation (B27) holds, but it gains some zero or negative eigenvalues if the condition fails. □

As a useful special case, when $N = 1$, J has only two eigenvalues, so one eigenvalue is determined by $\partial \dot{p}/\partial p$ while the sign of the second eigenvalue is determined by equation (B27): it is positive when the condition holds and zero or negative when it fails. When $N > 1$, we cannot determine exactly how many eigenvalues change sign when the condition fails, only that at least one must change sign. We do not analyze

[2] Also known as Engel's form or Titu's lemma, it is a consequence of the Cauchy-Schwarz inequality.

the eigenspaces at these equilibria further because we were unable to find closed-form solutions for the eigenvalues and eigenvectors of J_w for arbitrary r.

Note that the condition given in equation (B27) is equivalent to checking whether $\Lambda > 0$ using equation (B18) at the point $w = \mathbf{1}NR^*/\|r\|_1$ (for $R^* = R_1^*$ or R_2^*; here $\mathbf{1} = (1, \ldots, 1) \in \mathbb{R}^N$). In other words, if this condition holds, then the point at which the vector $\mathbf{1}$ intersects the hyperplanes of equilibria analyzed in Appendix B.1 is unstable, $\Lambda > 0$. Conversely, if this condition fails to hold, then the point at which $\mathbf{1}$ intersects the hyperplanes has $\Lambda \leq 0$.

For the symmetric model, $f_+(w) = w(1-w)$ and $f_-(w) = \alpha w(1-w)$. Consequently, at $w = \mathbf{0}$, J_w is the zero matrix, so the zero eigenvalue of J has multiplicity N. The final eigenvalue, with eigenvector $(0, \ldots, 0, 1)$, is equal to $\partial\dot{p}/\partial p$, which is identical to the expressions computed above for the additive model: at $p = 0$ and $p = 1$ it is negative, while at $p = R_2^*/(R_1^* + R_2^*)$ it is positive.

We do not fully analyze the zero eigenspaces at these critical points. However, note that the equation for $\dot{w}_i$ for the symmetric model is identical to the corresponding equation for the additive model multiplied by $w_i(1 - w_i)$, which does not change the sign. This means that the qualitative behavior of the system – towards or away from these equilibria – will be the same as in the case of the additive model, although the dynamics here are higher-order in w due to the $w_i(1 - w_i)$ term.

B.3 Extra Equilibria in the Symmetric Model

The symmetric model has a number of additional equilibria not present in the additive model. There are equilibria at $w = \mathbf{1}$ and either $p = 0$, 1, or $(R_2^* - \|r\|_1/N)/(R_1^* + R_2^* - 2\|r\|_1/N)$. More generally, for any partition of the index set $I \cup J \cup K = \{1, 2, \ldots, N\}$, there are equilibria at points w of the form

$$\begin{aligned} w_i &= 0 \text{ for } i \in I \\ w_j &= 1 \text{ for } j \in J \\ w_k = \tfrac{\tau(\alpha-1)\sum_{\ell\in I} r_\ell}{1-\tau(\alpha-1)\sum_{\ell\in K} r_\ell} &\text{ for } k \in K. \end{aligned} \tag{B29}$$

This requires that $\tau(\alpha-1)\sum_{\ell\in J\cup K} r_\ell < 1$ to ensure that $0 < w_k < 1$ if K is nontrivial. These points are equilibria when either $p = 0$, $p = 1$, or

$$p = \frac{R_2^* - \frac{1}{N}\left(\left(\sum_{\ell\in J} r_\ell\right) + w_k \sum_{\ell\in K} r_\ell\right)}{R_1^* + R_2^* - \frac{2}{N}\left(\left(\sum_{\ell\in J} r_\ell\right) + w_k \sum_{\ell\in K} r_\ell\right)}$$

where w_k is as above. We will focus our analysis only on the $w = \mathbf{1}$ equilibria, as most of our experiments employ $N = 1$ and the other equilibria require $N > 1$.

Proposition 5 *Suppose* $0 < R_1^*, R_2^* < \|r\|_1/N$. *If equation* (B27) *holds, then under the symmetric model the* $w = \mathbf{1}$, $p = 0$ *and* $w = \mathbf{1}$, $p = 1$ *equilibria have all positive eigenvalues, whereas if* $\tau(\alpha-1) > \|r\|_1^{-1}$, *then they are saddles with one positive eigenvalue and* N *negative eigenvalues. Meanwhile, the* $w = \mathbf{1}$, $p = (R_2^* - \|r\|_1/N)/(R_1^* + R_2^* - 2\|r\|_1/N)$ *equilibrium is a saddle with one negative eigenvalue and* N *positive eigenvalues if equation* (B27) *holds and if* $\tau(\alpha - 1) > \|r\|_1^{-1}$, *then it is stable.*

Proof The Jacobian at these equilibria can be computed as follows:

$$\frac{\partial \dot{w}_i}{\partial w_j} = -\left(pR_1^* + (1-p)R_2^* - \frac{\|r\|_1}{N}\right) r^{\text{dop}} \tau_{\text{dop}} \tau_{\text{eli}} \frac{\lambda}{N} \left(\tau(1-\alpha)\|r\|_1 + 1\right) r_i \delta_{ij},$$

$$\frac{\partial \dot{w}_i}{\partial p} = 0,$$

$$\frac{\partial \dot{p}}{\partial w_j} = \overline{\lambda}\beta r^{\text{dop}} \tau_{\text{dop}} p(1-p)(1-2p)\frac{r_j}{N},$$

$$\frac{\partial \dot{p}}{\partial p} = \overline{\lambda}\beta r^{\text{dop}} \tau_{\text{dop}} \Bigg((1-2p)\left(p\left(R_1^* - \frac{\|r\|_1}{N}\right) - (1-p)\left(R_2^* - \frac{\|r\|_1}{N}\right)\right) + p(1-p)\left(R_1^* + R_2^* - 2\frac{\|r\|_1}{N}\right)\Bigg).$$

Write the Jacobian matrix as

$$J = \begin{pmatrix} J_w & \mathbf{0} \\ \partial\dot{p}/\partial w & \partial\dot{p}/\partial p \end{pmatrix}$$

where

$$J_w = -\left(pR_1^* + (1-p)R_2^* - \frac{\|r\|_1}{N}\right) r^{\text{dop}} \tau_{\text{dop}} \tau_{\text{eli}} \frac{\lambda}{N} \left(\tau(1-\alpha)\|r\|_1 + 1\right) \operatorname{diag}(r).$$

The eigenvalues of J_w are multiples of the entries of r. We typically assume $r_i > 0$ for all i, and for these fixed points we assume $0 < R_1^*, R_2^* < \|r\|_1/N$. (If $\|r\|_1/N = R_1^*$ or R_2^* is allowed, J_w will be zero at either $p = 0$ or $p = 1$; for simplicity, we do not examine this case.) Recall that $\alpha \geq 1$. The signs of the eigenvalues are therefore determined by the same condition as that we found for the additive model, equation (B27): if $\tau(\alpha - 1) < \|r\|_1^{-1}$, then all eigenvalues of J_w are positive, while if $\tau(\alpha-1) > \|r\|_1^{-1}$, then all eigenvalues are negative.

The last eigenvalue (with eigenvector $(0,\ldots,0,1)$) equals $\partial\dot{p}/\partial p$:

$$\text{At } p = 0\text{:} \quad \frac{\partial \dot{p}}{\partial p} = -\overline{\lambda}\beta r^{\text{dop}} \tau_{\text{dop}} \left(R_2^* - \frac{\|r\|_1}{N}\right)$$

$$\text{At } p = \frac{R_2^* - \|r\|_1/N}{R_1^* + R_2^* - 2\|r\|_1/N}\text{:} \quad \frac{\partial \dot{p}}{\partial p} = \overline{\lambda}\beta r^{\text{dop}} \tau_{\text{dop}} \frac{(R_1^* - \|r\|_1/N)(R_2^* - \|r\|_1/N)}{R_1^* + R_2^* - 2\|r\|_1/N}$$

$$\text{At } p = 1\text{:} \quad \frac{\partial \dot{p}}{\partial p} = -\overline{\lambda}\beta r^{\text{dop}} \tau_{\text{dop}} \left(R_1^* - \frac{\|r\|_1}{N}\right).$$

Since $0 < R_1^*, R_2^* < \|r\|_1/N$, the first and third of these quantities are positive, while the second is negative. This, combined with our analysis of J_w, gives the stability conditions described above. □

B.4 Corticostriatal Model

Since D_+ and D_- depend on an incomplete gamma function applied to w, it is infeasible to analytically solve for most of the equilibria of the system when using the corticostriatal model. We can, however, identify the equilibria at $w = \mathbf{0}$, because at this point $D_+ = pR_1^* + (1-p)R_2^*$ (see equation (A12)) and so $D_- = 0$. It follows that, as in the additive and symmetric models, $w = \mathbf{0}$, $p = 0$, 1, and $R_2^*/(R_1^* + R_2^*)$ are equilibria. The Jacobian at these points can be computed as follows:

$$\frac{\partial \dot{w}_i}{\partial w_j} = r^{\text{dop}} \tau_{\text{dop}} \tau_{\text{eli}} \frac{\lambda}{N} \left(D_+|_{w=\mathbf{0}} \left(\tau r_i r_j + \delta_{ij} r_i\right) - D_+|_{w=\mathbf{0}}\, \tau r_i r_j \right),$$

$$
\begin{aligned}
\frac{\partial \dot{w}_i}{\partial p} &= 0, \\
\frac{\partial \dot{p}}{\partial w_j} &= \overline{\lambda}\beta r^{\mathrm{dop}}\tau_{\mathrm{dop}} p(1-p)(1-2p)\frac{r_j}{N}, \\
\frac{\partial \dot{p}}{\partial p} &= \overline{\lambda}\beta r^{\mathrm{dop}}\tau_{\mathrm{dop}}\left(p(2-3p)R_1^* + (1-p)(3p-1)R_2^*\right)
\end{aligned}
$$

for $p = 0,\ 1,\ R_2^*/(R_1^* + R_2^*)$. At $w = \mathbf{0}$, $D_+ = pR_1^* + (1-p)R_2^*$ and $D_- = 0$, so J_w is given by:

$$
J_w = \left(pR_1^* + (1-p)R_2^*\right) r^{\mathrm{dop}}\tau_{\mathrm{dop}}\tau_{\mathrm{eli}}\frac{\lambda}{N}\left(\tau r r^T + \mathrm{diag}(r)\right).
$$

All other terms are the same as in Appendix B.2. It is easy to see that J_w is positive definite, as $\mathrm{diag}(r)$ is positive definite (since $r_i > 0$ for all i) and rr^T is positive semidefinite, so their sum must be positive definite. We can calculate $\partial\dot{p}/\partial p$ as in Appendix B.2 to get that the $w = \mathbf{0}, p = 0$ and $w = \mathbf{0}, p = 1$ equilibria have Jacobians with one only one negative eigenvalue and the rest positive, while for the $w = \mathbf{0}, p = R_2^*/(R_1^* + R_2^*)$ equilibrium, the Jacobian has only positive eigenvalues, so only the unstable eigenspace is nontrivial.

As can be seen from Figure 7, however, the corticostriatal model also has additional equilibria at nonzero values of w, for which we do not have closed-form expressions.

Appendix C Action Selection Setting

C.1 Averaged Model

Our derivation of the averaged model for the action selection setting resembles the derivation in Appendix A, with some important modifications. Recall that in this setting, an action is selected in the window $[t_{\mathrm{dop}} - T_{\mathrm{del}} - T_{\mathrm{win}},\ t_{\mathrm{dop}} - T_{\mathrm{del}}]$, by measuring the relative activity in each channel as defined in equation (A3), and then spiking activity in the non-selected channel is completely suppressed until the next action selection window, while activity in the selected channel is reduced by a factor of a_{sel}. To model this scenario, we need to separately consider the cases where action A_1 or A_2 is selected. Using equation (8), we have:

$$
\mathbb{E}[D \mid A = A_k] = R_k^* - \left(R_1^*\,\mathbb{E}[p] + R_2^*(1 - \mathbb{E}[p])\right)
$$

and the probability of picking action A_k, when averaged over instantiations of the spike trains, is given by $\mathbb{E}[p]$ or $1 - \mathbb{E}[p]$ for $k = 1$ or 2, respectively, as defined in equation (9). When computing $\dot{w}^j$, we only include the $\mathbb{E}[D \mid A = A_j]$ term, since when $k \neq j$ all activity is suppressed, and we replace r with the rescaled firing rate vector $a_{\mathrm{sel}} r$, since activity in the selected channel is reduced by this factor. (Note that we use the original r in the computation of $\mathbb{E}[p]$, since activity is not suppressed during the action selection window.) Finally, we assume, as we did in the value estimation setting, that T_{del} is large, so that activity in the action selection window will be approximately

independent of the eligibility values of the weights at the later time when dopamine is released.

With the modifications described above, an analogous calculation to that made in the value estimation setting gives the following equation for the average weight drift for the additive and symmetric models:

$$\begin{aligned}\dot{w}^j = (-1)^{j-1}(R_1^* - R_2^*)\,\mathbb{E}[p](1-\mathbb{E}[p])r^{\text{dop}}\tau_{\text{dop}}\tau_{\text{eli}}\frac{\lambda}{N} \\ \times\left(a_{\text{sel}}^2\tau\langle w^j, r\rangle\Delta f(w^j)\odot r + a_{\text{sel}}f_+(w^j)\odot w^j\odot r\right).\end{aligned} \tag{C30}$$

For the corticostriatal model, we must take into account that the form of the weight update equation depends on the sign of the dopamine signal as described in equation (5). If $R_1^* > R_2^*$, as we typically assume, then the dopamine signal will always be greater than or equal to zero when action A_1 is selected, and less than or equal to zero when A_2 is selected. Since channel 1 is only active when A_1 is selected and is suppressed when A_2 is selected, the equation for $\dot{w}^1$ only includes the terms for positive dopamine, and likewise the equation for $\dot{w}^2$ only includes the terms for negative dopamine. We therefore obtain the following system of equations:

$$\begin{aligned}\dot{w}^1 &= |R_1^* - R_2^*|\,\mathbb{E}[p](1-\mathbb{E}[p])r^{\text{dop}}\tau_{\text{dop}}\tau_{\text{eli}}\tfrac{\lambda}{N} \\ &\quad\times\left(a_{\text{sel}}^2\tau\langle w^1, r\rangle(1-(1+\alpha)w^1)\odot r + a_{\text{sel}}(1-w^1)\odot w^1\odot r\right), \\ \dot{w}^2 &= |R_1^* - R_2^*|\,\mathbb{E}[p](1-\mathbb{E}[p])r^{\text{dop}}\tau_{\text{dop}}\tau_{\text{eli}}\tfrac{\lambda}{N} \\ &\quad\times\left(a_{\text{sel}}^2\tau\langle w^2, r\rangle(1-(1+\alpha)w^2)\odot r - a_{\text{sel}}\alpha w^2\odot w^2\odot r\right).\end{aligned} \tag{C31}$$

Note that each term in these equations has the same sign as the corresponding term in equation (A13) because $D_+ \geq 0$ and $D_- \leq 0$. If $R_1^* < R_2^*$, then we simply swap the equations.

C.2 Dynamics

For both the additive and symmetric models in the action selection setting, each $\dot{w}^j$ has a zero at $w^j = \mathbf{0}$. The symmetric model has zeros at $w^j = \mathbf{1}$, due to the $1-w$ terms in f_+, f_-, as well as the combinations of these points: $w^1 = \mathbf{0}, w^2 = \mathbf{1}$ and $w^1 = \mathbf{1}, w^2 = \mathbf{0}$. Additionally, when $N > 1$ each $\dot{w}^j$ for the symmetric model has zeros at the same points found in the value estimation case, specified in equation (B29); we do not analyze these points further.

In general, we cannot directly compute the Jacobians at these equilibria because of the $\mathbb{E}[p]$ terms, which depend on w via equation (7). However, it is still possible to describe the dynamics in many cases. We now prove the theorem stated in the main text:

Theorem 1 *Suppose that for the additive or symmetric models in the action selection setting, the following condition is satisfied:*

$$a_{sel}\tau(\alpha - 1) < \frac{1}{\|r\|_1}, \tag{13}$$

and suppose $R_1^* > R_2^*$. Then the $w^1 = w^2 = \mathbf{0}$ equilibrium is a saddle that is repelling along the w^1 coordinates and attracting along the w^2 coordinates. For the symmetric model, $w^1 = \mathbf{1}, w^2 = \mathbf{0}$ is an attractor, $w^1 = \mathbf{0}, w^2 = \mathbf{1}$ is a repelling equilibrium, and $w^1 = w^2 = \mathbf{1}$ is a saddle that is attracting along the w^1 coordinates and repelling along the w^2 coordinates.

Proof We will determine the dynamics by studying the simplified system,

$$\dot{u} = a_{\mathrm{sel}}^2 \tau \langle u, r \rangle (1 - \alpha) r + a_{\mathrm{sel}} u \odot r. \tag{C32}$$

Since $\mathbb{E}[p] > 0$, the dynamical equations for $\dot{w}^1$ and $\dot{w}^2$ for both additive and symmetric models (equation (C30)) are equal to substituting w for u in equation (C32) and multiplying by a function that is either always greater than or equal to zero or always less than or equal to zero, depending on the sign of $(-1)^j (R_1^* - R_2^*)$. It follows that any zero of $\dot{u}$ is also an equilibrium of the original system, and that the direction of flow near an equilibrium is either identical or reversed, again depending on the sign of $(-1)^j (R_1^* - R_2^*)$.

The Jacobian of this system at $u = \mathbf{0}$ is given by:

$$J_u = a_{\mathrm{sel}}^2 \tau (1 - \alpha) r r^T + a_{\mathrm{sel}} \operatorname{diag}(r).$$

This formula closely resembles equation (B28), the formula for J_w in Appendix B.2. Calculations analogous to those performed there show that J_u is positive definite if and only if equation (13) holds.

It follows that for both the additive and symmetric models, if equation (13) holds and $R_1^* > R_2^*$, then near the equilibrium $w^1 = w^2 = \mathbf{0}$ we will have $w_i^1 \geq 0$ and $w_i^2 \leq 0$ for all i.

To determine the stability of the symmetric model at $w^j = \mathbf{1}$, we use the system

$$\dot{v} = \left(a_{\mathrm{sel}}^2 \tau \langle v, r \rangle (1 - \alpha) r + a_{\mathrm{sel}} v \odot r \right) \odot (1 - v). \tag{C33}$$

Its Jacobian at $v = \mathbf{1}$ is:

$$J_v = -\left(a_{\mathrm{sel}}^2 \tau (1 - \alpha) \|r\|_1 + a_{\mathrm{sel}} \right) \operatorname{diag}(r).$$

The eigenvalues of J_v are multiples of the entries of r, and it can readily be seen that if equation (13) holds (and $r_i > 0$ for all i, as we generally assume), then J_v is negative definite.

It follows that if equation (13) holds and $R_1^* > R_2^*$, then the point $\mathbf{1}$ is attracting for w^1 and repelling for w^2. Combining these results, we see that under the symmetric model, $w^1 = \mathbf{1}, w^2 = \mathbf{0}$ is an attractor, $w^1 = \mathbf{0}, w^2 = \mathbf{1}$ is a repelling equilibrium, and $w^1 = w^2 = \mathbf{1}$ is a saddle which is attracting along the w^1 coordinates and repelling along the w^2 coordinates. □

For the additive model, this leads to relatively simple dynamics. If $R_1^* > R_2^*$, w^1 will generally grow until it hits the boundary at $\mathbf{1}$, while w^2 will approach the origin. For the symmetric model, the dynamics are similar when $N = 1$, except that $\mathbf{1}$ is a genuine zero of $\dot{w}^1$, rather than just an accumulation point caused by clipping the weights to $[0, 1]$. When $R_1^* < R_2^*$ all these relations are reversed; thus in both settings the dynamics promote selecting the more rewarding action. When $N > 1$, though, the dynamics under the symmetric model may be more complex due to the presence of other equilibria.

For the corticostriatal model, in addition to the zeros at $w^j = \mathbf{0}$, there is also a set of zeros that differ for the w^1 and w^2 equations. Assuming $R_1^* > R_2^*$, so that

equation (C31) is used, the points w^{1*} and w^{2*} defined below are zeros of $\dot{w}^1$ and $\dot{w}^2$, respectively:

$$w_i^{1*} = \frac{a_{\text{sel}}\tau\|r\|_1 + 1}{a_{\text{sel}}\tau\|r\|_1(1+\alpha) + 1} \quad \forall i,$$
$$w_i^{2*} = \frac{a_{\text{sel}}\tau\|r\|_1}{a_{\text{sel}}\tau\|r\|_1(1+\alpha) + \alpha} \quad \forall i.$$

We cannot directly compute the Jacobians at these points because of the $\mathbb{E}[p]$ terms. However, we can determine their stability by considering the reduced system

$$\begin{aligned}\dot{w}^1 &= a_{\text{sel}}^2\tau\langle w^1, r\rangle(1-(1+\alpha)w^1)\odot r + a_{\text{sel}}(1-w^1)\odot w^1\odot r,\\ \dot{w}^2 &= a_{\text{sel}}^2\tau\langle w^2, r\rangle(1-(1+\alpha)w^2)\odot r - a_{\text{sel}}\alpha w^2\odot w^2\odot r.\end{aligned}$$

This system is produced by dividing the terms in equation (C31) by strictly positive functions, so it has the same equilibria with identical stability properties. The Jacobians at $w_i^1 = w^{1*}, w_i^2 = w^{2*}$ are:

$$\begin{aligned}J^1 &= a_{\text{sel}}^2\tau\left(1-(1+\alpha)w^{1*}\right)rr^T + \left(a_{\text{sel}}(1-2w^{1*}) - a_{\text{sel}}^2\tau\|r\|_1(1+\alpha)w^{1*}\right)\text{diag}(r)\\ &= -\frac{a_{\text{sel}}^2\alpha\tau}{a_{\text{sel}}\tau\|r\|_1(1+\alpha)+1}rr^T - a_{\text{sel}}\frac{(1+\alpha)\left(a_{\text{sel}}\tau\|r\|_1\right)^2 + 2a_{\text{sel}}\tau\|r\|_1 + 1}{a_{\text{sel}}\tau\|r\|_1(1+\alpha)+1}\text{diag}(r),\\ J^2 &= a_{\text{sel}}^2\tau\left(1-(1+\alpha)w^{2*}\right)rr^T - \left(2a_{\text{sel}}\alpha w^{2*} + a_{\text{sel}}^2\tau\|r\|_1(1+\alpha)w^{2*}\right)\text{diag}(r)\\ &= -\frac{a_{\text{sel}}^2\alpha\tau}{a_{\text{sel}}\tau\|r\|_1(1+\alpha)+1}rr^T - a_{\text{sel}}\frac{(1+\alpha)\left(a_{\text{sel}}\tau\|r\|_1\right)^2 + 2\alpha a_{\text{sel}}\tau\|r\|_1}{a_{\text{sel}}\tau\|r\|_1(1+\alpha)+\alpha}\text{diag}(r)\end{aligned}$$

which are both negative definite matrices. It follows that both w^{1*} and w^{2*} are attractors for their respective equations, so that w^1 converges to w^{1*} and w^2 converges to w^{2*}. Since $w^{1*} > w^{2*}$, the more rewarding action, A_1, will be favored over A_2. The $R_1^* < R_2^*$ case can be treated similarly.

Appendix D Single Eligibility Trace

We now revisit the question of whether to use a single eligibility trace summing up both positive (corresponding to pre-before-post spike pairs) and negative (post-before-pre) contributions, as is done in [21], or to use two different traces for the positive and negative components, as we do elsewhere in the paper. We had several reasons for focusing on models with two different eligibility traces. One was analytical convenience: the use of two traces is necessitated by the assumption, made by [24, 26] as well as in this paper, that the contributions to the weight changes made by individual spike pairs sum independently, a natural assumption that greatly simplifies analysis. With only one eligibility trace, different spike pairs may cancel each other out, rendering this independence assumption invalid. We therefore cannot derive averaged forms of the single-trace models like we did for the two-trace models. A second justification for the focus on two-trace models is that there is experimental evidence suggesting that

the brain in fact uses two different traces, one for LTP and one for LTD [37], at least in cortical pyramidal neurons.

Nonetheless, for modeling purposes, it is important to compare the performance of the single-trace model to the model two-trace model that we have considered. We therefore test a single-trace version of our model that replaces equation (3) with

$$\frac{dE_i}{dt} = \rho^{\text{post}}(t)A_i^{\text{pre}}(t) - \gamma\rho_i^{\text{pre}}(t)A^{\text{post}}(t) - \frac{1}{\tau_{\text{eli}}}E_i(t) \tag{D34}$$

where $\gamma \geq 1$ is a scaling parameter controlling the strength of negative eligibility terms relative to positive terms. The single-trace differential equation for the weights in the additive and symmetric cases is given by

$$\frac{dw_i}{dt} = \begin{cases} \lambda D(t) f_+(w_i(t))E_i(t) & \text{if } E_i(t) \geq 0 \\ \lambda D(t) f_-(w_i(t))E_i(t) & \text{if } E_i(t) < 0. \end{cases} \tag{D35}$$

and for the corticostriatal model is given by

$$\frac{dw_i}{dt} = \begin{cases} \lambda D(t)(1 - w_i(t))E_i(t) & \text{if } D(t)E_i(t) \geq 0 \\ \lambda D(t)\alpha w_i(t)E_i(t) & \text{if } D(t)E_i(t) < 0. \end{cases}$$

The single-trace version of the corticostriatal model is largely equivalent to the model described in [21], although they use different scaling factors and time constants for pre- and postsynaptic activity.

One important characteristic of the single-trace versions of the additive and symmetric models (equation (D35)) is that they are largely insensitive to variations in α. This insensitivity arises because $E_i(t)$ is usually positive, since presynaptic spikes directly cause postsynaptic spikes after a delay of ϵ and not vice versa, which tilts the balance to favor positive eligibility. Hence, the α-dependent f_- term is only rarely used. The α parameter is therefore not an effective way of adjusting the relative strengths of the positive and negative components of the eligibility trace. This observation motivates the introduction of the parameter γ in equation (D34) to provide a better means of controlling the relative strengths of the two components in the single-trace models. The single-trace corticostriatal model is still sensitive to α because it depends on the sign of the product $D(t)E_i(t)$, rather than just $E_i(t)$, so the term with α will have an impact when $E_i(t) > 0$ and $D(t) < 0$.

We show simulations of the single-trace models in the action selection setting in Figures 12 and 13 (without and with contingency switching), and in the value estimation setting in Figures 14 and 15. In the action selection setting, we see that the behavior of the additive and symmetric models is qualitatively very similar to their behavior in the two-trace settings (compare Figure 12 to Figure 3 and Figure 13 to Figure 5), with varying γ having a very similar effect to varying α in the two-trace versions. The corticostriatal model also behaves similarly when γ is small, but as γ increases its behavior deviates significantly from that in the two-trace setting, which can lead to worse performance in some cases: the flow in Figure 12i is away from the

optimal weight values $w^1 = 1, w^2 = 0$, while in Figure 3i it converges to (w^{1*}, w^{2*}) which, while not optimal, still leads to the correct action being selected.

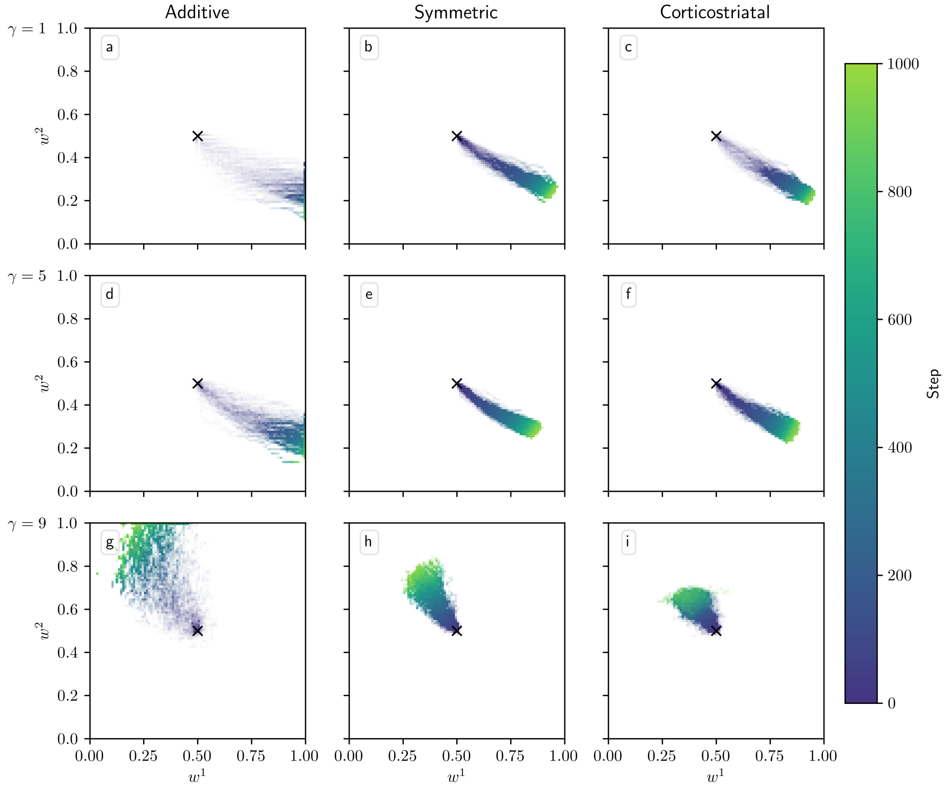

Fig. 12 Distribution of w^1 and w^2 over time in the action selection setting for single-trace models as γ is varied. Columns show the additive (a, d, g), symmetric (b, e, h), and corticostriatal (c, f, i) models. γ is varied across rows: (a-c) $\gamma = 1$; (d-f) $\gamma = 5$; (g-i) $\gamma = 9$. We use $\alpha = 1$ and $w_{\text{init}} = 0.5$ here, marked by the "×" in each plot. As we do not have averaged forms of the single-trace dynamics we do not include vector fields or fixed points here

In the value estimation setting, we again see that the additive and symmetric models behave qualitatively similarly across the single-trace and two-trace versions of the dynamics (compare Figure 14 to Figure 7 and Figure 15 to Figure 9). Like in the action selection setting, as γ increases the corticostriatal model's behavior deviates from that in the two-trace setting. In Figure 14f and i, this leads to convergence to somewhat different equilibria; in Figure 15i and l, this actually leads to improved performance over the two-trace version seen in Figure 9i and l (although as we noted previously, performance at this task is quite sensitive to the learning rates λ and $\overline{\lambda}$, and better choices for their values may lead to better performance).

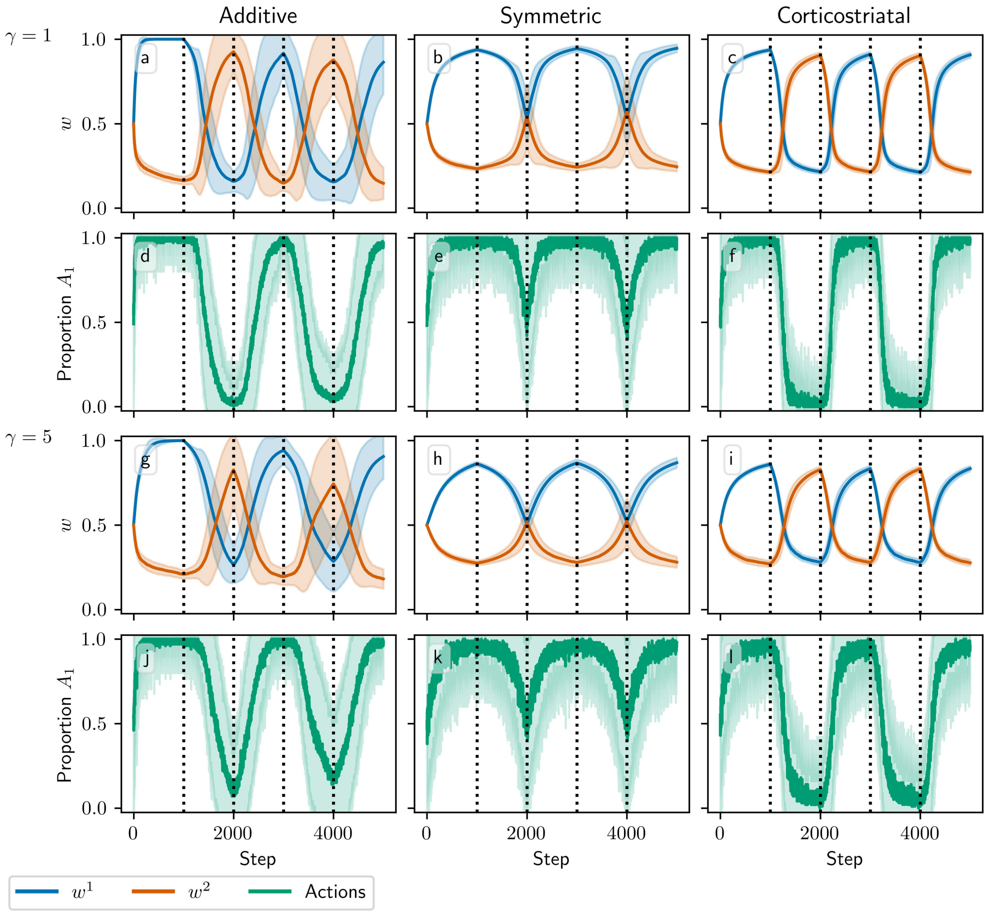

Fig. 13 Model performance in the action selection setting with contingency switching for single-trace models as γ is varied. Plots show weights (a-c, g-i) and probability of taking the correct action (d-f, j-l) versus time for the additive (a, d, g, j), symmetric (b, e, h, k), and corticostriatal (c, f, i, l) models. γ is varied across rows: (a-f) $\gamma = 1$; (g-l) $\gamma = 5$. We use $\alpha = 1$ and the other parameters are the same as those used in Figure 5

Overall, using a single eligibility trace does not seem to significantly improve performance in most cases, it makes the dynamics much more difficult to analyze, it complicates the weight equations for the additive and symmetric cases, and it requires tuning the artificial parameter γ to capture effects of $\alpha > 1$. On the other hand, it does halve the number of eligibility trace equations that need to be simulated.
